# Hyperconnectivity of prefrontal cortex to amygdala projections in a mouse model of macrocephaly/autism syndrome

**DOI:** 10.1038/ncomms13421

**Published:** 2016-11-15

**Authors:** Wen-Chin Huang, Youjun Chen, Damon T. Page

**Affiliations:** 1 Department of Neuroscience, The Scripps Research Institute, Jupiter, Florida 33458, USA; 2 The Doctoral Program in Chemical and Biological Sciences, The Scripps Research Institute, USA

## Abstract

Multiple autism risk genes converge on the regulation of mTOR signalling, which is a key effector of neuronal growth and connectivity. We show that mTOR signalling is dysregulated during early postnatal development in the cerebral cortex of germ-line heterozygous *Pten* mutant mice (*Pten*^*+/−*^), which model macrocephaly/autism syndrome. The basolateral amygdala (BLA) receives input from subcortical-projecting neurons in the medial prefrontal cortex (mPFC). Analysis of mPFC to BLA axonal projections reveals that *Pten*^*+/−*^ mice exhibit increased axonal branching and connectivity, which is accompanied by increased activity in the BLA in response to social stimuli and social behavioural deficits. The latter two phenotypes can be suppressed by pharmacological inhibition of S6K1 during early postnatal life or by reducing the activity of mPFC–BLA circuitry in adulthood. These findings identify a mechanism of altered connectivity that has potential relevance to the pathophysiology of macrocephaly/autism syndrome and autism spectrum disorders featuring dysregulated mTOR signalling.

## Abstract (web summary)

Dysregulation of mTOR signaling has been implicated in autism spectrum disorders. Here authors show that hyperconnectivity and hyperactivity of the mPFC–BLA circuitry in *Pten* ^+/−^ mice underlies their social impairments, and that reducing mTORC1 signaling during early postnatal development rescues these deficits.

The mechanistic target of rapamycin (mTOR) is a serine/threonine kinase that integrates environmental cues (for example, growth factors and energy status) to control cell growth and proliferation^1^. mTOR interacts with several proteins to form at least two distinct multiprotein complexes: mTOR complex 1 (mTORC1) and mTOR complex 2 (mTORC2). When activated, mTORC1 promotes protein synthesis mainly by phosphorylation of ribosomal protein S6 kinase (S6K) and eukaryotic initiation factor 4E-binding protein (eIF4E-BP)^2^. Phosphorylation of eIF4E-BP by mTORC1 releases eIF4E from binding to eIF4E-BP, forming the eIF4F complex to initiate cap-dependent protein translation^1^. Phosphorylation of ribosomal protein S6 (rpS6) by S6K correlates with the translational efficiency of messenger RNAs containing a tract of oligopyrimidine in the 5′UTR, named 5′TOP messenger RNA^3^. Phosphorylated rpS6 (p-S6) is a readout for mTORC1 signalling, and has been shown to regulate cell size^4^. Moreover, studies in the mammalian nervous system have shown that mTOR-S6K signalling regulates neuronal soma size, dendritic arborization, axonal growth and connectivity^5^,^6^,^7^.

Numerous lines of evidence implicate dysregulated mTOR signalling in the pathogenesis of autism spectrum disorder (ASD) and related neurodevelopmental disorders. Genes impinging on the PI3K-Akt-mTOR pathway, for example, *phosphatase and tensin homologue* (*PTEN*)*, TSC1/2, NF1* and *FMR1,* are highly represented amongst ASD risk genes identified to date^8^. Elevated mTOR signalling in the cerebral cortex has been reported in postmortem samples from individuals with autism^9^, and several animal models of risk genes acting in this pathway also show altered mTOR signalling in the brain^10^,^11^,^12^,^13^,^14^.

*PTEN* encodes a phosphatase that is a negative regulator of the PI3K-Akt-mTOR pathway^15^. Germ-line heterozygous mutations in *PTEN* are found in ∼7–17% individuals with autism and macrocephaly^16^ (head circumference >2 s.d.'s above normal), generally reduce PTEN protein levels^17^,^18^, and cause macrocephaly/autism syndrome (OMIM #605309) in an autosomal dominant manner. A mouse model that approximates the germ-line heterozygous *Pten* (*Pten*^*+/−*^) mutations found in macrocephaly/autism syndrome displays a dynamic pattern of macroscale brain overgrowth due to altered proliferation and allocation of cell types, as well as selective impairments in ASD-relevant behaviours, particularly social behaviour^19^,^20^,^21^,^22^. The widespread macroscale brain overgrowth, together with selective behavioural impairments, suggests that cell types and circuits underlying social behaviour may be more vulnerable or less adaptable to desynchronized patterns of growth caused by *Pten* mutations^20^.

This raises a critical problem for understanding the neurobiology of autism and developing effective treatments: how do mutations in general regulators of growth, such as *PTEN* and other members of the PI3K-Akt-mTOR pathway, lead to the relatively selective behavioural and cognitive symptoms of ASD? To answer this question, it is necessary to understand the spatiotemporal pattern of activity of the PI3K-Akt-mTOR pathway in the developing brain and how it is impacted by mutations in ASD risk genes at the level of structural and functional connectivity in key circuits underlying ASD-relevant behaviours.

Here we have mapped the activity of mTORC1, using p-S6 as a readout, in the developing brains of normal and germ-line *Pten*^*+/−*^ mice. We report that p-S6 levels are unexpectedly stable in the developing cerebral cortex of germ-line *Pten*^*+/−*^ mice, with the exception of a discreet window during early postnatal life. Among the cell types that display particularly high levels of p-S6 are developing subcortical-projecting layer V neurons. We report that these neurons are hypertrophic in germ-line *Pten*^*+/−*^ mice and that cortical–subcortical connectivity is altered in these animals. Connectivity between the medial prefrontal cortex (mPFC) and the basolateral amygdala (BLA) is a particular focus of this study, as both of these areas are implicated in the processing of social information^23^ and autism pathophysiology^24^. Furthermore, structural and functional connectivity between these areas is well conserved between rodents and humans^25^. Our findings are consistent with the novel hypothesis that dysregulated mTORC1 signalling during a critical period of development alters neuronal connectivity, including mPFC–BLA projections, and social behaviour.

## Results

### mTOR activity is dysregulated in *Pten* mutant mice

**Figure 1:**
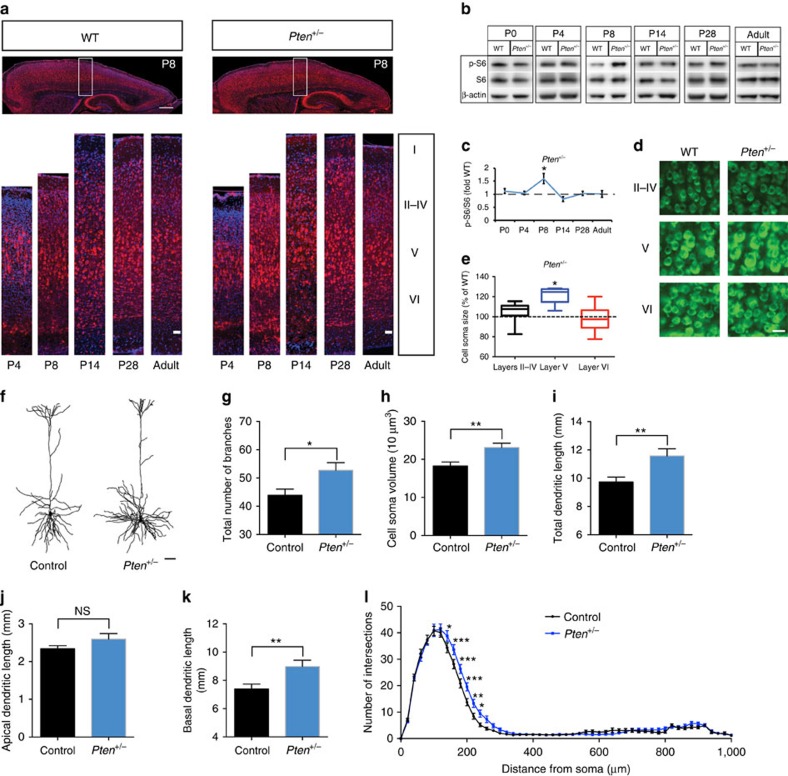
Characterization of mTORC1 activity and neuronal growth in *Pten* mutant mice. (**a**) Images showing phospho-S6 (p-S6), a readout for mTORC1 activity, immunostaining in the cerebral cortex of postnatal day 8 (P8) WT and *Pten*^*+/−*^ mice. Phospho-S6 (red), DAPI (blue) and scale bar, 500 μm. Representative somatosensory cortex with p-S6 immunostaining in P4, P8, P14, P28, and adult WT and *Pten*^*+/−*^ mice. Phospho-S6 (red), DAPI (blue), and scale bar, 50 μm. (**b**) Representative images of western blot for p-S6, total S6 and β-actin in WT and *Pten*^*+/−*^ whole cerebral cortex at P0, P4, P8, P14, P28 and adulthood. (**c**) Quantification of p-S6 levels relative to total S6, expressed as fold WT, in the whole cerebral cortex of WT and *Pten*^*+/−*^ mice at P0, P4, P8, P14, P28 and adult. Independent-sample *t* tests were used. *P<0.05. *N*=5 animals per genotype for each time point. (**d**) Representative images of fluorescent Nissl stain in somatosensory cortical layers II–VI of WT and *Pten*^*+/−*^ mice at P14. Scale bar, 25 μm. (**e**) Quantification of cell soma size in somatosensory cortical layers II–VI of WT and *Pten*^*+/−*^ mice at P14, expressed as per cent of WT. Independent-sample *t* tests were used. *P<0.05. *N*=6 animals in each genotype. (**f**) Representative images of reconstructed layer V neurons in the somatosensory cortex of P14 *Etv1-CreERT2; Ai14* (Control) and *Etv1-CreERT2; Ai14; Pten*^*+/−*^ (*Pten*^*+/−*^) mice. Scale bar, 50 μm. (**g**–**k**) Quantification of total number of branches (**g**), cell soma volume (**h**), and total (**i**), apical (**j**) and basal (**k**) dendrite length of layer V neurons in P14 control and *Pten*^*+/−*^ mice. Independent-sample *t* tests were used. *P<0.05, and **P<0.01 and NS indicates no significant difference. *N*=25 neurons/6 animals per genotype. (**l**) Sholl analysis of somatosensory cortex layer V neurons in P14 control and *Pten*^*+/−*^ mice. Two-way ANOVA with Bonferroni correction, *F*(50, 2,448)=2.174, *P*<0.0001 (interaction); *P<0.05, **P<0.01, and ***P<0.001. *N*=25 neurons/6 animals each genotype. All mice used in this figure were male.

To understand whether the laminar distribution of p-S6 in the cerebral cortex is altered in *Pten*^*+/−*^ mice, we performed immunohistochemistry for p-S6 and compared the *Pten*^*+/−*^ pattern of p-S6 to that of wild type (WT) mice at postnatal day 4 (P4), P8, P14, P28, and adulthood. To our surprise, we found that the spatiotemporal distribution of p-S6 was not grossly altered in the developing cerebral cortex of *Pten*^*+/−*^ mice (Fig. 1a). To compare levels of p-S6 between WT and *Pten*^*+/−*^ mice, we ran western blots on whole cortices from these animals across developmental time points. We found that p-S6 levels were unexpectedly similar between WT and *Pten*^*+/−*^ mice at all stages examined with the exception of P8, when there was more p-S6 detected in the *Pten*^*+/−*^ cortex as compared with WT (Fig. 1b,c). This indicates that p-S6 levels are transiently dysregulated during early postnatal life (between P4 and P14) in *Pten*^*+/−*^ mice.

In carrying out immunohistochemistry for p-S6 within the early postnatal cerebral cortex, the non-uniform laminar distribution of p-S6 in WT, as well as *Pten*^*+/−*^, mice was notable. Plotting the distribution of p-S6 immunoreactivity across layers in the somatosensory cortex at P8 revealed that p-S6 levels are graded, with strongest signal in layer V and moderate signal in layers II/III and VI (Supplementary Fig. 1A). Measuring neuronal soma size and p-S6 immunoreactivity at P8, we found that p-S6 immunoreactivity is correlated with soma size (Supplementary Fig. 1B). Moreover, within cortical layer V, subcortical-projecting neurons have greater soma size and dendritic complexity than callosal-projecting neurons^26^,^27^, and we observed that p-S6 immunoreactivity is greater in CTIP2^+^ neurons, which are subcortical-projecting neurons^28^, than in SATB2^+^ neurons, which are callosal-projecting neurons^29^ (Supplementary Fig. 1C). Examining other brain areas, we found that during early postnatal development p-S6 was generally enriched in larger cell types (cell soma ≥20 μm in diameter), for example, Purkinje cells in the cerebellum, layer V corticospinal motor neurons (LV neurons), dopaminergic neurons (DA neurons) and noradrenergic neurons (NA neurons; Supplementary Fig. 1D). In contrast, minimal *P*-S6 was generally detected during development in smaller cell types (cell soma ≤10 μm in diameter), for example, medium spiny neurons, hippocampal granule cells and cerebellar granule cells (Supplementary Fig. 1D). We interpret this expression pattern as consistent with p-S6 being a positive regulator of cell/neuronal size^4^,^5^,^6^,

Given that we detected a change in the amplitude, but not the spatiotemporal distribution, of p-S6 in the developing cortex, we predicted that loss of one copy of *Pten* (that is, *Pten*^*+/−*^) might particularly impact cell types with high levels of p-S6 in WT animals. As an initial test of this, we examined the size of neurons across layers in the somatosensory cortex. We selected this region because of its relatively well-defined laminar organization within the mouse cerebral cortex. Using fluorescent Nissl staining, we detected increased soma size in layer V neurons, but not in layers II–IV or VI neurons, in P14 *Pten*^*+/−*^ mice as compared with WT mice (Fig. 1d,e). This indicates that the growth of cell types enriched for p-S6 (for example, layer V neurons), as reflected by cell soma size, is desynchronized relative to neurons with lower levels of p-S6 (for example, layers II–IV or VI neurons) in *Pten*^*+/−*^ mice.

As we observed enrichment of p-S6 in developing subcortical-projecting layer V neurons, amongst other cell types, and we detected a pronounced change in cell soma size in layer V neurons in *Pten*^*+/−*^ mice, we decided to examine these as a representative of cell types whose growth is impacted by *Pten* haploinsufficiency. To understand whether overgrowth of layer V neurons is restricted to the cell soma or also occurs in neurites, we examined dendritic structure. We made use of a genetic reporter for layer V neurons (*Etv1-CreERT2; Ai14*)^30^,^31^ and measured dendritic tree size of these cells in P14 *Pten*^*+/−*^ mice (*Etv1-CreERT2; Ai14*; *Pten*^*+/−*^). We found that, relative to controls, layer V neurons in *Pten*^*+/−*^ mice exhibited increased cell soma volume and dendritic complexity (Fig. 1f–l). Thus, changes in neuronal soma size in layer V neurons caused by *Pten* haploinsufficiency correlate with changes in neurite growth.

To explore whether dysregulated neurodevelopmental mTORC1 signalling might be a point of convergence across multiple mouse models of autism risk genes, we performed these analyses in another mouse model of an autism risk gene, *Fmr1*^*−/y*^. We found that, similar to *Pten*^*+/−*^ mice, the spatiotemporal distribution of p-S6 in the cerebral cortex is not grossly altered, and p-S6 levels are elevated at P14 as compared with WT, but not other ages (Supplementary Fig. 2A–C), indicating that cortical mTORC1 signalling is transiently elevated during early postnatal life. Measuring cell soma size across cortical layers, we found that layer V neurons are enlarged in P14 *Fmr1*^*−/y*^ mice as compared with WT mice (Supplementary Fig. 2D,E). Consistent with overgrowth in layer V neurons, dendritic tree size is also slightly greater in P14 *Etv1-CreERT2*; *Fmr1*^*−/y*^mice as compared to control littermates (Supplementary Fig. 2F–L). This suggests that multiple autism risk genes may converge on dysregulated neurodevelopmental mTORC1 signalling and desynchronized neuronal growth.

### Early inhibition of S6K1 corrects deficits in *Pten* mutants

**Figure 2:**
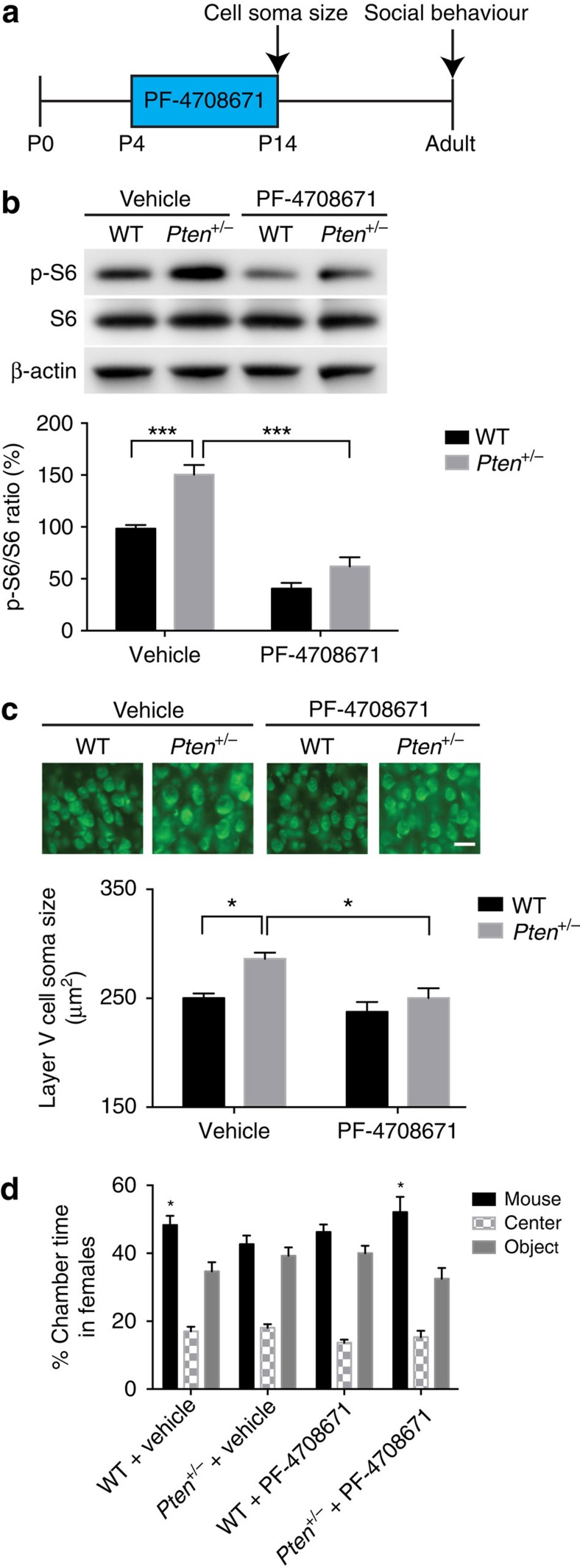
Inhibition of S6K1 during development corrects cellular and behavioural deficits in *Pten* mutant mice. (**a**) Diagram showing time line of PF-4708671 treatment and analysis of layer V cell soma size and social behaviour. (**b**) Representative images of western blot for p-S6, total S6 and β-actin in the cerebral cortex of P8 WT and *Pten*^*+/−*^ mice receiving either vehicle or PF-4708671. Quantification of p-S6 levels relative to total S6, expressed as per cent of WT. Two-way ANOVA and Tukey *post hoc* tests were used. *F*(1,16)=4.488, *P*=0.05 (interaction), *F*(1,16)=102.2, *P*<0.001 (drug), *F*(1,16)=25.54, *P*<0.001 (genotype); ***P<0.001. *N*=5 animals per group. (**c**) Representative images of fluorescent Nissl staining in the layer V somatosensory cortex and quantification of layer V cell soma size in WT and *Pten*^*+/−*^ mice receiving either vehicle or PF-4708671 from P4 to P14. Planned comparisons revealed a significant difference between WT and *Pten*^*+/−*^ mice receiving vehicle, and a significant difference between vehicle and PF-4708671-treated *Pten*^*+/−*^ mice, with two-way ANOVA showing main effects of drug and genotype. *F*(1,16)=2.541, *P*=0.131 (interaction), *F*(1,16)=10.96, *P*<0.01 (drug), *F*(1,16)=10.96, *P*<0.01 (genotype). *P<0.05. *N*=5 animals per group. Scale bar, 25 μm. (**d**) Per cent time WT and *Pten*^*+/−*^ mice receiving either vehicle or PF-4708671 from P4 to P14 spent in each chamber during the three-chamber social approach test. Two-way ANOVA and Sidak's *post hoc* tests were used. *F*(3,82)=3.334, *P*=0.0234 (interaction), *F*(3,82)=0.2417, *P*=0.867 (genotype) and *F*(1,82)=28.87, *P*<0.001 (chamber time). *P<0.05. *N*=11 WT receiving vehicle, 13 *Pten*^*+/−*^ receiving vehicle, 11 WT receiving PF-4708671, and 10 *Pten*^*+/−*^ receiving PF-4708671. All mice used in this figure were female.

The observation that p-S6 levels are transiently dysregulated in the cerebral cortex of P8 *Pten*^*+/−*^ mice led us to speculate that this could represent a critical period where normalization of p-S6 levels may suppress the overgrowth of layer V neurons and behaviour deficits in these animals. To test this possibility, we treated *Pten*^*+/−*^ mice and WT controls with the S6K1 inhibitor PF-4708671 (ref. 32), injected daily from P4 to P14 (Fig. 2a). We found that systemic PF-4708671 treatment reduced elevated p-S6 in the cerebral cortex and suppressed the increased soma size of layer V neurons in the somatosensory cortex of P14 *Pten*^*+/−*^ mice (Fig. 2b,c). The most robust and consistent behavioural phenotype we have observed in *Pten*^*+/−*^ mice is deficits in the three-chamber social approach test in adult females^20^,^21^,^22^. Adult male *Pten*^*+/−*^ mice, in contrast to females, display variable phenotypes in this assay that are highly sensitive to testing conditions^20^,^21^ (Supplementary Fig. 3), so three-chamber social approach testing in this study focused on female animals. We found that PF-4708671 treatment in the P4–P14 developmental window is sufficient to reverse the three-chamber social approach deficit present in vehicle-treated adult female *Pten*^*+/−*^ mice (Fig. 2d). We also observed that WT mice receiving PF-4708671 treatment displayed social approach deficits, consistent with a previous report of reduced social interaction in WT mice receiving rapamycin treatment beginning at 5–6 weeks of age^10^. Thus, we speculate that an optimal level of mTORC1 signalling is required during development for normal social behaviour, with too much or too little activity resulting in similar social behavioural deficits, in line with a previously proposed model of bidirectional control of protein synthesis and connectivity in ASD^33^. Taken together, these results point to a developmental window in early postnatal life in which social behaviour is sensitive to modulation of mTOR/S6K1 signalling and normalization of p-S6 levels results in lasting correction of the three-chamber social approach deficits in *Pten*^*+/−*^ mice.

In addition to the core symptoms of ASD, some individuals with germ-line heterozygous *PTEN* mutations also have deficits in working memory^17^. To explore whether germ-line *Pten*^*+/−*^ mice also show working memory deficits and whether this phenotype may be responsive to developmental PF-4708671 treatment, we assessed spatial working memory using the non-matching to sample T-maze test (Supplementary Fig. 4A)^34^. Vehicle and PF-4708671-treated WT and *Pten*^*+/−*^ mice of both sexes showed normal performance in this test (Supplementary Fig. 4B,C), indicating that the particular aspect of working memory probed by the non-matching to sample T-maze test is not altered in these animals.

### Neuronal hyperconnectivity in *Pten* mutants

**Figure 3:**
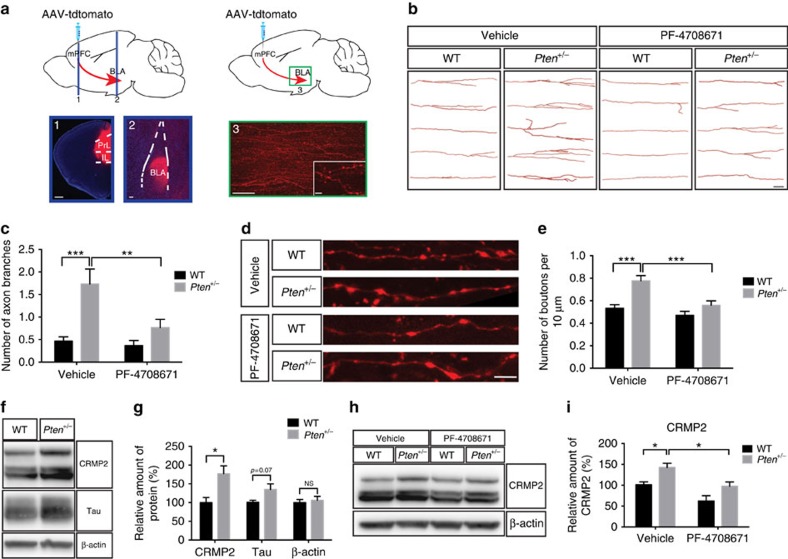
Hyperconnectivity of mPFC to BLA circuit in *Pten* mutant mice is corrected by inhibition of S6K1 during development. (**a**) Images showing tdTomato expression in coronal sections of mPFC and basal lateral amygdala (BLA), and sagittal section showing mPFC-originating axon terminals and synaptic boutons in the BLA. Scale bar, 500 μm (1), 100 μm (2), 50 μm (3) and 5 μm (right bottom image of (3)). (**b**,**c**) Representative images (**b**) and quantification (**c**) of reconstructed mPFC axon terminals in the BLA of adult WT and *Pten*^*+/−*^ mice receiving either vehicle or PF-4708671 from postnatal day 4 (P4) to P14. Scale bar, 50 μm. Two-way ANOVA and Tukey *post hoc* tests were used, *F*(1,116)=4.548, *P*<0.05 (interaction), *F*(1,116)=6.89, *P*<0.01 (drug), *F*(1,116)=16.82, *P*<0.001 (genotype); **P<0.01, ***P<0.001. *N*=30 axons from 6 mice in each group. (**d**,**e**) Representative images (**d**) and quantification (**e**) of mPFC–BLA synaptic boutons in adult WT and *Pten*^*+/−*^ mice receiving either vehicle or PF-4708671 from P4 to P14. Scale bar, 5 μm. Two-way ANOVA and Tukey *post hoc* tests were used, *F*(1,116)=4.094, *P*<0.05 (interaction), *F*(1,116)=13.30, *P*<0.001 (drug), *F*(1,116)=18.79, *P*<0.001 (genotype); ***P<0.001. *N*=30 axon fragments from 6 mice in each group. (**f**,**g**) Representative images (**f**) of western blot for CRMP2, Tau and β-actin in the cerebral cortex of P8 WT or *Pten*^*+/−*^ mice. (**g**) Quantification of CRMP2 and Tau protein levels relative to β-actin, and total β-actin levels, expressed as percentage of WT. Independent-sample *t* tests were used. *P<0.05. *N*=5 animals per genotype. (**h**,**i**) Representative images (**h**) of western blot for CRMP2 and β-actin in the cerebral cortex of P8 WT or *Pten*^*+/−*^ mice injected with either vehicle or PF-4708671 from P4 to P8. (**i**) Quantification of CRMP2 protein levels relative to β-actin. Planned comparisons revealed a significant difference between WT and *Pten*^*+/−*^ mice receiving vehicle, and a significant difference between vehicle and PF-4708671-treated *Pten*^*+/−*^ mice, with two-way ANOVA showing main effects on drug and genotype. *F*(1,16)=0.089, *P*=0.77 (interaction), *F*(1,16)=16.82, *P*<0.001 (drug), *F*(1,16)=14.24, *P*<0.01 (genotype). *P<0.05. *N*=5 animals in each group. All mice used in this figure were female.

Considering that layer V neurons are the primary output neurons of the cerebral cortex, we speculated that cortical–subcortical connectivity between areas important for social behaviour might be altered in these animals. A circuit that is particularly interesting in this context involves projections from the mPFC to the BLA (see Fig. 3a). Both of these areas are implicated in the processing of social information^23^ and autism pathophysiology^24^. Thus, we formulated the hypothesis that dysregulated mTORC1 signalling during a critical period of development alters neuronal connectivity, including mPFC–BLA projections, and social behaviour.

To test this hypothesis, we first confirmed that BLA-projecting mPFC neurons are hypertrophic in *Pten*^*+/−*^ mice (Supplementary Fig. 5A–C), consistent with our observation of layer V neuron hypertrophy in somatosensory cortex (Fig. 1e). Furthermore, we found that protein synthesis and p-S6 levels are dysregulated in the prefrontal cortex of P8 *Pten*^*+/−*^ mice, indicating dysregulated mTORC1 activity (Supplementary Fig. 5D–G). We next injected reporter-expressing (tdTomato) adeno-associated virus (AAV) into the mPFC of adult female mice and visualized axon terminal branching and synaptic boutons within the BLA (Fig. 3a). We found that axons originating from the mPFC in vehicle-treated *Pten*^*+/−*^ mice displayed excessive branching and synaptic boutons within the BLA, as compared with vehicle-treated WT mice, and that PF-4708671 treatment during development (P4–P14) was sufficient to reverse this phenotype in *Pten*^*+/−*^ mice (Fig. 3b–e). These results indicate that mPFC–BLA circuitry is hyperconnected in *Pten*^*+/−*^ mice, and that this phenotype is sensitive to early postnatal PF-4708671 treatment.

As an initial exploration into the molecular mechanism by which dysregulated mTORC1 signalling might lead to altered axon branching and structural connectivity, we measured levels of CRMP2 (collapsin response mediated protein 2) and Tau (microtubule associated protein), both of which are regulated by mTORC1 signalling and have been shown to influence axon growth^35^,^36^. We observed a significant increase in CRMP2 levels and a trend towards increased Tau levels in P8 *Pten*^*+/−*^ cerebral cortex (Fig. 3f,g). Actin remodelling is a regulator of axon growth, and homozygous *Pten* mutations can alter actin levels^37^. Thus, we measured β-actin levels and did not observe any differences between WT and *Pten*^*+/−*^ cortex (Fig. 3g), indicating that this is a valid loading control and that heterozygous *Pten* mutations do not grossly alter β-actin levels, unlike homozygous *Pten* mutations. Since the excessive axon branching in *Pten*^*+/−*^ mice was suppressed by pharmacological inhibition of S6K1 during early postnatal life, we suspected that this effect could be due to the inhibition of S6K1-dependent translation of CRMP2 (ref. 36). To test this possibility, we measured CRMP2 levels in the P8 cerebral cortex of *Pten*^*+/−*^ and WT control mice that received PF-4708671 treatment from P4 to P8, by western blot. We found that PF-4708671 treatment suppressed elevated CRMP2 in *Pten*^*+/−*^ mice (Fig. 3h,i). Thus, normalization of excessive axon branching in *Pten*^*+/−*^ mice by PF-4708671 treatment correlates with normalization of CRMP2 levels in these animals.

### Hyperactivity in the mPFC and BLA of *Pten* mutant mice

**Figure 4:**
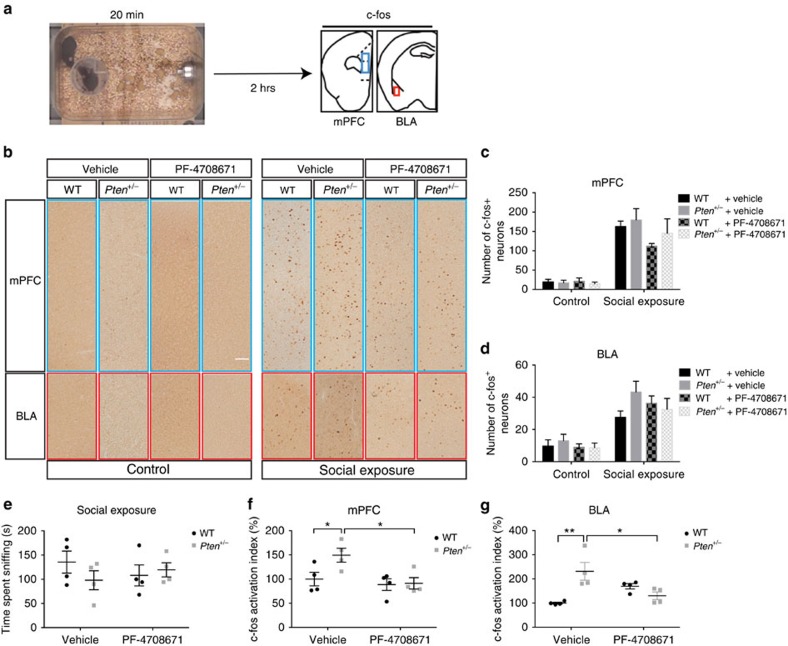
mPFC and BLA hyperactivity in *Pten* mutant mice is corrected by inhibition of S6K1 during development. (**a**) Diagram showing the workflow of social exposure and c-fos staining. Adult mice were exposed to a novel mouse for 20 min, perfused 2 h later, and then underwent c-fos staining to measure neuronal activity in the mPFC and BLA. (**b**–**d**) Representative images (**b**) and quantification of c-fos staining in the mPFC (**c**) and the basal lateral amygdala (BLA) (**d**) of control or social exposed WT and *Pten*^*+/−*^ mice receiving either vehicle or PF-4708671 from postnatal day 4 (P4) to P14. Scale bar, 100 μm. *N*=4 animals in each group. (**e**) Graph showing social interaction time, reflected as time spent sniffing, in WT and *Pten*^*+/−*^ mice receiving either vehicle or PF-4708671 from P4 to P14. *N*=4 animals in each group. (**f**,**g**) Quantification of c-fos activation index (number of c-fos^+^ neurons/time spend sniffing) in the mPFC (**f**) and BLA (**g**) of social exposed WT and *Pten*^*+/−*^ mice receiving either vehicle or PF-4708671 from P4 to P14. Planned comparisons revealed a significant difference between WT and *Pten*^*+/−*^ mice receiving vehicle, and a significant difference between vehicle and PF-4708671-treated *Pten*^*+/−*^ mice. For (**f**) two-way ANOVA revealed a main effect on drug. *F*(1,12)=3.194, *P*=0.099 (interaction), *F*(1,12)=7.069, *P*<0.05 (drug), *F*(1,12)=3.947, *P*=0.07 (genotype). For (**g**) two-way ANOVA revealed main effects on genotype and interaction. *F*(1,12)=16.8, *P*<0.01 (interaction), *F*(1,12)=0.571, *P*=0.464 (drug), *F*(1,12)=4.901, *P*<0.05 (genotype). *P<0.05 and **P<0.01. *N*=4 animals in each group. All mice used in this figure were female.

To test if neuronal activity in response to a social cue is altered in *Pten*^*+/−*^ mice, we measured the number of c-fos^+^ neurons in the mPFC and BLA in response to exposure to a novel stimulus mouse (Fig. 4a). Previous work has shown that social exposure induces c-fos activation in the mPFC and BLA, and that the number of c-fos^+^ cells in these two regions correlates with the amount of time engaged in social interaction^38^. Consistent with this, we found that exposure to a novel social stimulus results in a robust induction of c-fos^+^ cells in the mPFC and BLA of both WT and *Pten*^*+/−*^ adult female mice (Fig. 4b–d) and that the number of c-fos^+^ cells in these two regions correlated with the duration each mouse spent sniffing the novel stimulus mouse (Supplementary Fig. 6A). Although the total number of c-fos^+^ cells in the mPFC and BLA is not altered in *Pten*^*+/−*^ mice compared with WT mice (Fig. 4c,d), we found that social interaction time, as reflected by time spent sniffing, varied between and within genotype during the social exposure period (Fig. 4e). Because the induction of c-fos^+^ cells in mPFC and BLA is dependent on the amount of social interaction time, we calculated a c-fos activation index (number of c-fos^+^ neurons/time mouse engaged in sniffing; Supplementary Fig. 6B). We found that the c-fos activation index is elevated in the mPFC and BLA of vehicle-treated *Pten*^*+/−*^ mice. Furthermore, we found that the elevated c-fos activation index in the mPFC and BLA is normalized in *Pten*^*+/−*^ mice receiving PF-4708671 treatment during early postnatal life (P4–P14; Fig. 4f,g). We confirmed this result in a separate cohort of mice in which the animals were allowed to sniff a novel stimulus mouse to a fixed criterion of 30 s, thus ensuring equivalent amounts of exposure to the social cue across subjects. In this cohort, we found that c-fos^+^ cells in the mPFC and BLA are elevated in *Pten*^*+/−*^ relative to WT mice, and this phenotype is rescued by PF-4708671 treatment during early postnatal life (Supplementary Fig. 7). Thus, with equivalent social exposure, there are more c-fos^+^ cells in the mPFC and BLA of *Pten*^*+/−*^ mice, and this phenotype can be normalized by suppression of S6K1 during development.

### Adult S6K1 inhibition does not rescue *Pten* mutant deficits

**Figure 5:**
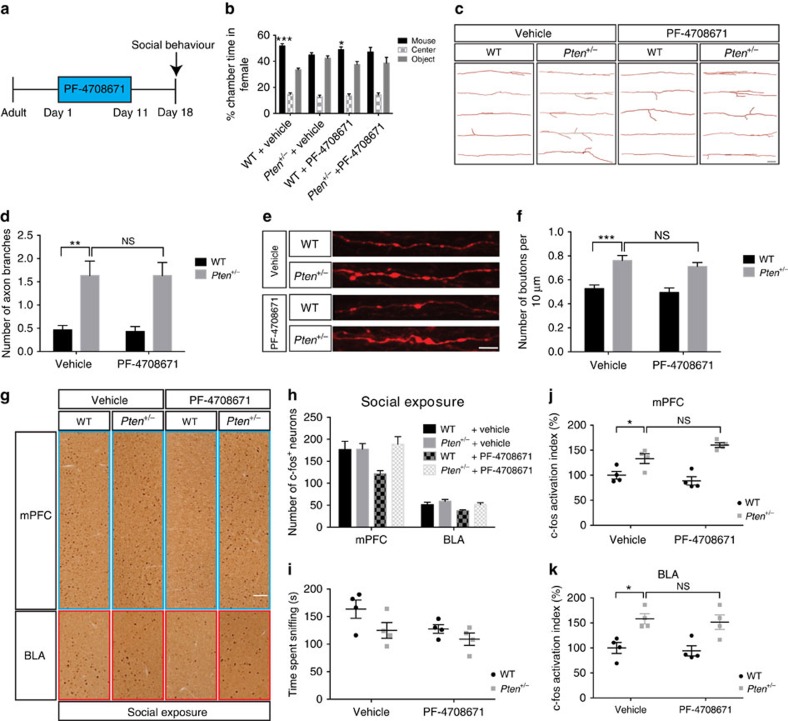
Inhibition of S6K1 in adulthood does not rescue cellular and behavioural deficits in *Pten* mutant mice. (**a**) Diagram showing time line of PF-4708671 treatment and testing of social behaviour. (**b**) Graph showing per cent time mice spent in each chamber. Two-way ANOVA and Sidak's *post hoc* tests were used. *F*(3,88)=3.59, *P*=0.0168 (interaction), *F*(3,88)=0.0573, *P*=0.9819 (genotype), and *F*(1,88)=33.20, *P*<0.001 (chamber time). *N*=12 WT receiving vehicle, 12 *Pten*^*+/−*^ receiving vehicle, 13 WT receiving PF-4708671 and 11 *Pten*^*+/−*^ receiving PF-4708671. (**c**,**d**) Representative images (**c**) and quantification (**d**) of reconstructed mPFC axon terminals in the BLA. Scale bar, 50 μm. Planned comparisons revealed a significant difference between WT and *Pten*^*+/−*^ mice receiving vehicle, with two-way ANOVA showing a main effect on genotype. *F*(1,116)=0.006, *P*=0.94 (interaction), *F*(1,116)=0.006, *P*=0.94 (drug), *F*(1,116)=28.54, *P*<0.001 (genotype). *N*=30 axons from 6 mice in each group. (**e**,**f**) Representative images (**e**) and quantification (**f**) of mPFC–BLA synaptic boutons. Scale bar, 5 μm. Planned comparisons revealed a significant difference between WT and *Pten*^*+/−*^ mice receiving vehicle, with two-way ANOVA showing a main effect on genotype. *F*(1,116)=0.066, *P*=0.798 (interaction), *F*(1,116)=1.301, *P*=0.256 (drug), *F*(1,116)=40.33, *P*<0.001 (genotype). *N*=30 axon fragments from 6 mice in each group. (**g**,**h**) Representative images (**g**) and quantification (**h**) of c-fos staining in the mPFC and BLA of social exposed mice. Scale bar, 100 μm. *N*=4 animals in each group. (**i**) Graph showing time mice spent sniffing, reflecting social interaction time. *N*=4 animals in each group. (**j**,**k**) Quantification of c-fos activation index (number of c-fos^+^ neurons/time spend sniffing) in the mPFC (**j**) and BLA (**k**). Planned comparisons revealed a significant difference between WT and *Pten*^*+/−*^ mice receiving vehicle. For (**j**) two-way ANOVA revealed main effects on genotype and interaction. *F*(1,12)=5.964, *P*<0.05 (interaction), *F*(1,12)=1.008, *P*=0.335 (drug), *F*(1,12)=44.45, *P*<0.001 (genotype). For (**k**) two-way ANOVA revealed a main effect on genotype. *F*(1,12)=0.002, *P*=0.967 (interaction), *F*(1,12)=0.287, *P*=0.602 (drug), *F*(1,12)=24.87, *P*<0.001 (genotype). For all panels, *P<0.05, **P<0.01, ***P<0.001 and NS indicates not significantly different. *N*=4 animals in each group. All mice used in this figure were female adult mice.

We next asked whether mPFC–BLA connectivity and social phenotypes in *Pten*^*+/−*^ mice might be responsive to PF-4708671 treatment in adulthood, when cortical circuitry has reached a mature state. To address this issue, we treated adult *Pten*^*+/−*^ mice with the equivalent dosage and duration of PF-4708671 as that of developmental experiments and tested social behaviour 7 days after the final injection of PF-4708671 (Fig. 5a). We found that the three-chamber social approach deficit is not corrected in *Pten*^*+/−*^ mice treated with PF-4708671 in adulthood (Fig. 5b). This result prompted us to investigate mPFC–BLA structural connectivity and c-fos reactivity in these mice. We found that excessive axon branching and increased density of synaptic boutons in mPFC to BLA projections was not rescued in *Pten*^*+/−*^ mice receiving PF-4708671 treatment in adulthood (Fig. 5c–f). The elevated c-fos activation index in the mPFC and BLA in response to social exposure was also not rescued in *Pten*^*+/−*^ mice treated with PF-4708671 in adulthood (Fig. 5g–k). Taken together, our PF-4708671 treatment results indicate that there is a developmental critical period in which pharmacological suppression of S6K1 can cause a lasting rescue of mPFC–BLA hyperconnectivity, excessive c-fos reactivity and three-chamber social approach deficits in *Pten*^*+/−*^ mice.

### Hyperactivity of mPFC-BLA circuitry in *Pten* mutants

**Figure 6:**
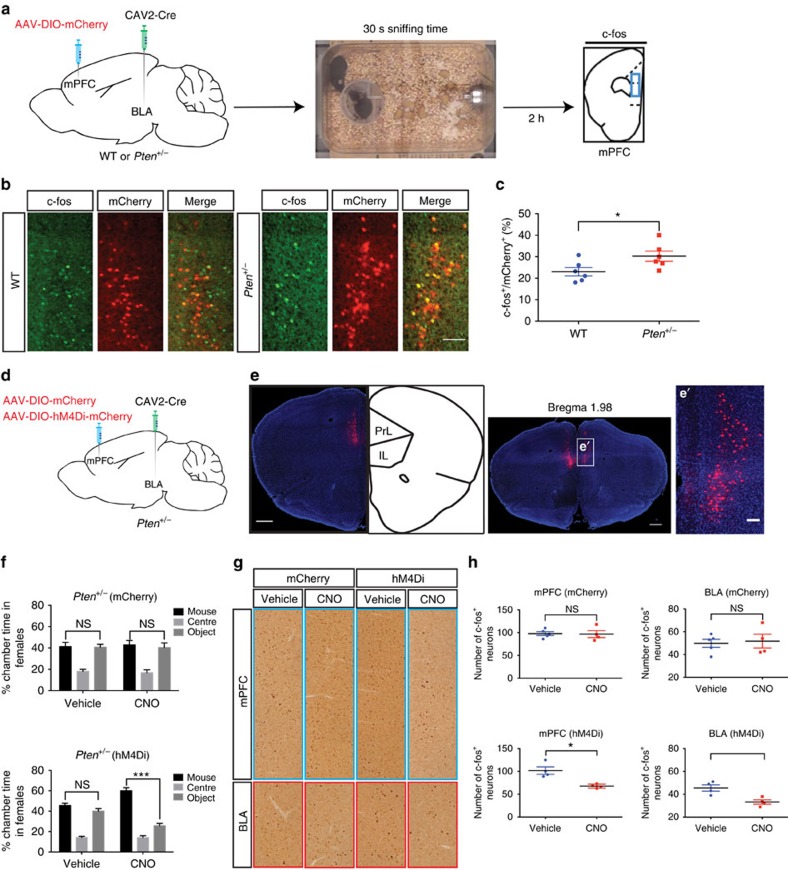
Reducing activity in the mPFC to BLA projections corrects social behavioural deficits in *Pten* mutant mice. (**a**) Diagrams showing experimental design. To label BLA-projecting mPFC neurons, CAV2-Cre was injected into the BLA, and AAV-DIO-mCherry was injected into the mPFC. Social exposure was controlled for 30 s sniffing time. (**b**) Representative images of mCherry (red) and c-fos (green) double labelling in the mPFC of WT and *Pten*^*+/−*^ mice. Scale bar, 100 μm. (**c**) Quantification of the percentage of c-fos^+^ neurons within the mCherry^+^ neuronal population. Independent-sample *t* tests were used. *P<0.05. *N*=6 animals per genotype. (**d**) Diagrams showing the injection of CAV2-Cre into the BLA and AAV-DIO-mCherry or AAV-DIO-hM4Di-mCherry into the mPFC of *Pten*^*+/−*^ mice. (**e**) Images showing reporter (mCherry) expression in mPFC. PrL: prelimbic cortex. IL: infralimbic cortex, with magnified images in (**e**′). Scale bar, 500 μm (left and middle) and 100 μm (right). (**f**) Per cent time spent in each chamber during the three-chamber social approach test. *N*=8 mice in *Pten*^*+/−*^ (hM4Di) group and 9 mice in *Pten*^*+/−*^ (mCherry) group. Two-way ANOVA and Sidak's *post hoc* tests were used. For mCherry group, *F*(1,32)=0.0569, *P*=0.813 (interaction), *F*(1,32)=0.02535, *P*=0.8745 (treatment) and *F*(1,32)=0.1798, *P*=0.6744 (chamber time). For hM4Di group, *F*(1,28)=32.40, *P*<0.001 (interaction), *F*(1,28)=0.00068, *P*=0.9794 (treatment) and *F*(1,28)=63.12, *P*<0.001 (chamber time). ***P<0.001 and NS indicates not significant difference. (**g,h**) Images (**g**) and quantification (**h**) of c-fos staining in the mPFC and BLA of social exposed *Pten*^*+/−*^ mice that expressed either hM4Di plus mCherry or mCherry alone in BLA-projecting mPFC neurons and received either vehicle or CNO injections. Scale bar, 100 μm. *N*=4 mice in hM4Di expressing *Pten*^*+/−*^ mice receiving either vehicle or CNO, *N*=5 mice in vehicle injected, and 4 mice in CNO injected, mCherry expressing *Pten*^*+/−*^ mice. Independent-sample *t* tests were used, *P<0.05. All mice used in this figure were female.

To test whether *Pten*^*+/−*^ mice exhibit hyperactive mPFC–BLA circuitry in response to social cues, we used c-fos expression as a measure of neuronal activity in BLA-projecting mPFC neurons in WT and *Pten*^*+/−*^ mice. To label BLA-projecting mPFC neurons, we injected an AAV vector carrying the reporter mCherry, expressed in a Cre-dependent manner (double-floxed inverted open reading frame) into the mPFC, and a retrograde-transporting canine adenovirus carrying Cre recombinase (CAV2-Cre) into the BLA of adult female WT and *Pten*^*+/−*^ mice (Fig. 6a). Each subject mouse was allowed to sniff a novel stimulus mouse to a fixed criterion of 30 s so that each subject mouse engaged in an approximately equal amount of social interaction. We found that a greater percentage of BLA-projecting mPFC neurons (mCherry^+^ neurons) were c-fos^+^ in *Pten*^*+/−*^ mice as compared with WT mice (Fig. 6b,c), suggesting that mPFC–BLA circuitry is hyperactive in response to social exposure in *Pten*^*+/−*^ mice.

To test whether decreasing activity in mPFC to BLA projections may normalize three-chamber social approach deficits in *Pten*^*+/−*^ mice, we reduced excitability in mPFC neurons projecting to the BLA using retro-DREADD (Designer Receptor Exclusively Activated by Designer Drugs) technology^39^. An AAV vector carrying hM4Di and mCherry expressed in a Cre-dependent manner was bilaterally injected into the mPFC, and a CAV2-Cre vector was bilaterally injected into the BLA of adult female *Pten*^*+/−*^ mice (Fig. 6d,e). Clozapine n-oxide (CNO) or vehicle was injected into these mice, and we then tested social behaviour using the three-chamber social approach assay. We found that the three-chamber social approach deficit present in vehicle injected hM4Di expressing *Pten*^*+/−*^ mice or vehicle and CNO injected mCherry expressing *Pten*^*+/−*^ mice was corrected in CNO injected hM4Di expressing *Pten*^*+/−*^ mice (Fig. 6f).

We next measured c-fos^+^ cells in the mPFC and BLA of these mice in response to sniffing a novel stimulus mouse to a fixed criterion of 30 s. We found that the number of c-fos^+^ cells in the mPFC and BLA in response to social exposure was reduced in CNO injected compared with vehicle injected hM4Di expressing *Pten*^*+/−*^ mice, and we found no change in the number of c-fos^+^ cells in either vehicle or CNO injected mCherry expressing *Pten*^*+/−*^ mice (Fig. 6g,h). We interpret these results as consistent with a causal relationship between hyperactivity in mPFC–BLA circuitry and three-chamber social approach deficits in *Pten*^*+/−*^ mice.

### Reducing mTOR activity corrects deficits in *Pten* mutant mice

**Figure 7:**
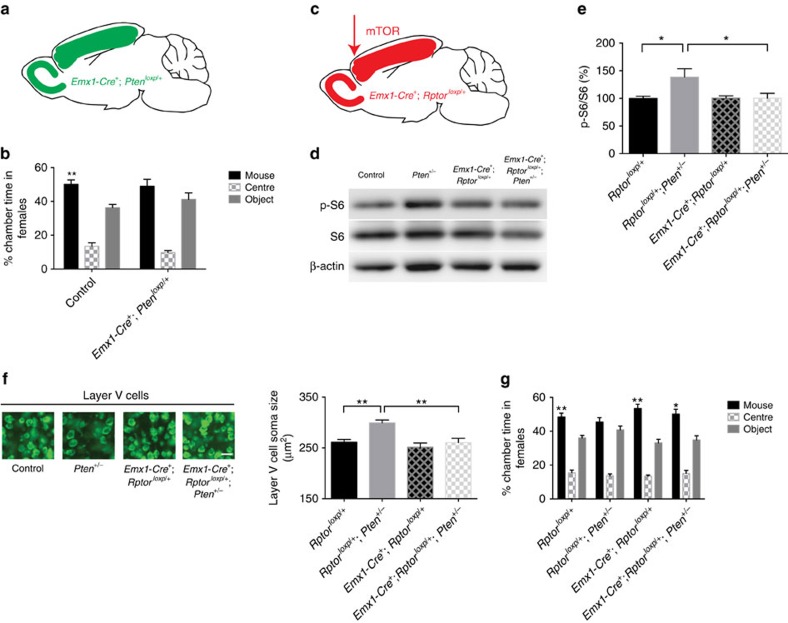
Suppression of mTOR activity corrects cellular and behavioural deficits in *Pten* mutant mice. (**a**) Schema showing the brain regions where one copy of *Pten* is deleted (green colour, primarily in the cerebral cortex). (**b**) Per cent time control and *Emx1-Cre*^*+*^*; Pten*^*loxp/+*^ mice spent in each chamber. Two-way ANOVA was used. *F*(1,56)=0.8877, *P*=0.3502 (interaction), *F*(1,56)=0.3242, *P*=0.5714 (genotype) and *F*(1,56)=11.16, *P*=0.0015 (chamber time). Paired *t* tests showed that control, but not *Emx1-Cre*^*+*^*; Pten*^*loxp/+*^, mice exhibited a significant preference for the social chamber. **P<0.01. *N*=15 animals in each genotype. (**c**) Schema showing the brain regions where one copy of *Rptor* is deleted in *Pten*^*+/−*^ mice (red colour, primarily in the cerebral cortex). (**d**,**e**) Representative images (**d**) of western blot for p-S6, total S6 and β-actin in the cerebral cortex of control (*Rptor*^*loxp/+*^), *Pten*^*+/−*^ (*Rptor*^*loxp/+*^*; Pten*^*+/−*^), *Emx1-Cre*^*+*^*; Rptor*^*loxp/+*^, and *Emx1-Cre*^*+*^*; Rptor*^*loxp/+*^*; Pten*^*+/−*^ mice. (**e**) Quantification of p-S6 levels relative to total S6, expressed as per cent of control. One-way ANOVA and Tukey *post hoc* tests were used. *F*(3,25)=4.153, *P*<0.05 (genotype), *P<0.05. *N*=8 control, 8 *Pten*^*+/−*^, 6 *Emx1-Cre*^*+*^*; Rptor*^*loxp/+*^, and 7 *Emx1-Cre*^*+*^*; Rptor*^*loxp/+*^*; Pten*^*+/−*^ mice. (**f**) Representative images of fluorescent Nissl staining in somatosensory cortical layer V, and quantification of layer V cell soma size in control (*Rptor*^*loxp/+*^), *Pten*^*+/−*^ (*Rptor*^*loxp/+*^*; Pten*^*+/−*^), *Emx1-Cre*^*+*^*; Rptor*^*loxp/+*^ and *Emx1-Cre*^*+*^*; Rptor*^*loxp/+*^*; Pten*^*+/−*^ mice. One-way ANOVA and Tukey *post hoc* tests were used. *F*(3,16)=9.183, *P*<0.001 (genotype), **P<0.01. *N*=5 animals per genotype. Scale bar, 25μm. (**g**) Per cent time mice spent in each chamber. Two-way ANOVA and Sidak's *post hoc* tests were used. *F*(3,60)=3.902, *P*=0.013 (interaction), *F*(3,60)=0.0842, *P*=0.9684 (genotype) and *F*(1,60)=60.65, *P*<0.001 (chamber time). *P<0.05, and **P<0.01. *N*=8 control (*Rptor*^*loxp/+*^), 8 *Pten*^*+/−*^ (*Rptor*^*loxp/+*^*; Pten*^*+/−*^), 10 *Emx1-Cre*^*+*^*; Rptor*^*loxp/+*^, and 8 *Emx1-Cre*^*+*^*; Rptor*^*loxp/+*^*; Pten*^*+/−*^ mice. All mice used in this figure were female.

To determine whether isolating *Pten*^*+/−*^ mutations to the cerebral cortex will lead to three-chamber social approach deficits, we generated conditional *Pten*^*+/−*^ mutant mice using *Emx1-Cre,* which is expressed primarily in cortical pyramidal cells throughout development^40^ (Fig. 7a). We used a Cre driver that is expressed broadly in the cortex, rather than a Cre driver that is restricted to subcortical-projecting pyramidal cells, because we do not know at present if the effects of *Pten*^*+/−*^ mutations on the development of this neuronal population are cell autonomous or non-cell autonomous. We have previously shown that *Emx1-Cre*^*+*^*; Pten*^*loxp/+*^ mice recapitulate the cortical overgrowth phenotype seen in germ-line *Pten*^*+/−*^ mice^19^. Testing social behaviour using three-chamber social approach test, we found that although adult female *Emx1-Cre*^*+*^*; Pten*^*loxp/+*^ mice did not display a significant difference in allocation of chamber time as compared with controls, a within-group analysis revealed a lack of significant difference in time spent in the stimulus mouse versus object chambers (Fig. 7b).

To test whether reducing mTORC1 signalling in the cerebral cortex of germ-line *Pten*^*+/−*^ mice is sufficient to correct three-chamber social approach deficits, we generated *Emx1-Cre*^*+*^; *Rptor*^*loxp/+*^; *Pten*^*+/−*^ mice (Fig. 7c). We found that dysregulated p-S6 in P8 *Pten*^*+/−*^ cerebral cortex was normalized in *Emx1-Cre*^*+*^; *Rptor*^*loxp/+*^; *Pten*^*+/−*^ mice (Fig. 7d,e). We measured cell soma size as a marker for layer V neuron structural changes in *Pten*^*+/−*^ mice and found that layer V neuronal hypertrophy was suppressed in *Emx1-Cre*^*+*^; *Rptor*^*loxp/+*^; *Pten*^*+/−*^ mice in adulthood (Fig. 7f). Moreover, the three-chamber social approach deficit present in adult female *Pten*^*+/−*^ mice was also corrected in *Emx1-Cre*^*+*^; *Rptor*^*loxp/+*^; *Pten*^*+/−*^ mice (Fig. 7g). Taken together, our results indicate that dysregulated mTOR signalling in the developing cerebral cortex contributes to social behavioural deficits in *Pten*^*+/−*^ mice. Future experiments will examine whether conditional *Pten*^*+/−*^ mutation or modulation of mTORC1 signalling specifically in subcortical-projecting pyramidal cells is sufficient to alter three-chamber social approach behaviour.

## Discussion

The genetic causes of ASD are heterogeneous. Studying a signalling pathway regulated by multiple autism risk genes at the level of circuit function will provide insights into common biological mechanisms underling ASD pathogenesis and pathophysiology. Here we have presented evidence that dysregulated mTOR signalling impacts the assembly of circuitry underlying social behaviour in germ-line *Pten*^*+/−*^ mice, a mouse model for macrocephaly/autism syndrome. We have mapped the spatiotemporal activity of mTORC1 signalling in developing mouse brain, and found that a subset of cells, including subcortical-projecting layer V neurons in the cerebral cortex, exhibit enrichment for p-S6, a readout for mTORC1 signalling. We further investigated cortical–subcortical connectivity, which is largely mediated by subcortical-projecting layer V neurons, between two brain areas that are implicated in the processing of social information and ASD pathophysiology, the mPFC and BLA. We show that *Pten*^*+/−*^ mice exhibit hyperconnectivity and hyperactivity of mPFC–BLA circuitry, excessive c-fos reactivity in response to social cues, and three-chamber social approach deficits. Correcting structural connectivity by treatment with a pharmacological inhibitor of S6K1 during early postnatal life or modulating neuronal activity by DREADDs in adulthood can suppress these phenotypes.

We find that *Pten*^*+/−*^ mice display non-uniform overgrowth across neuronal cell types in the developing cerebral cortex, indicating that the growth of some neuronal populations (for example, subcortical-projecting layer V neurons) is desynchronized relative to others. This finding has implications for information processing within cortical microcircuitry. *Fmr1*^*−/y*^ mice, which model fragile X syndrome, display a similar phenotype. One possible explanation for this finding is that mutations in genes that encode negative regulators of mTORC1 signalling, such as *Pten* and *Fmr1*, have the greatest effect on growth in cell types with high baseline levels of mTORC1 activity (for example, layer V neurons) because these cells are more dependent on negative regulation by repressive factors. It is worth contrasting the effects of heterozygous and homozygous *Pten* mutations in light of this explanation. Studies from *Pten* conditional knockout mice have shown that even relatively small neural cell types, such as hippocampal granule cells, exhibit overgrowth on conditional homozygous mutation of *Pten* (ref. 11). Indeed, we have found that conditional homozygous mutation of *Pten* leads to massively increased p-S6 levels and decreased cell density throughout the newborn cerebral cortex, consistent with widespread hypertrophy, whereas we did not observe this phenotype upon conditional heterozygous mutation of *Pten* (ref. 19). We attribute this to an effect of *Pten* dosage: homozygous mutation of *Pten* shifts a wide variety of neural cell types towards a pathological state, with strong upregulation of mTORC1 activity and hypertrophy. In contrast, most neural cell types appear to maintain more or less normal physiological levels of mTORC1 signalling upon heterozygous mutation of *Pten*.

The normal developmental trajectory of mTORC1 activity in the brain may help to explain our finding of transient changes in cortical p-S6 levels in early postnatal life in germ-line *Pten*^*+/−*^ and *Fmr1*^*−/y*^ mice. One study that examined mTORC1 signalling in the cerebral cortex of WT mice in both early postnatal life and adulthood found that mTORC1 signalling is most active during early postnatal life, especially the first two postnatal weeks^41^. Just as cell types with high baseline levels of mTORC1 signalling may be particularly vulnerable to the effects of dominant-acting mutations in genes that encode negative regulators of mTORC1 signalling, neurodevelopmental time windows of peak mTORC1 activity (for example, early postnatal life) might represent critical periods of vulnerability to such mutations, as well as targets for therapeutic intervention.

We found that in the mature brain, axonal projections from the mPFC to the BLA have excessive branches and synaptic boutons. Given that elevated cortical p-S6 and CRMP2, and rescue by S6K1 inhibitor treatment, all occur during the early postnatal life, we predict that the initial growth of subcortical-projecting neurons is over-exuberant in *Pten*^*+/−*^ mice. This would be consistent with reports of *Pten* and mTOR-S6K signalling acting as regulators of axonal growth^7^,^11^. Another factor that could be at play is reduced axonal pruning of subcortical-projecting neurons in *Pten*^*+/−*^ mice. This is an interesting possibility given that a recent study has reported reduced dendritic spine pruning in cortical layer V cells of individuals with ASD and in *Tsc2* mutant mice, with this phenotype resulting from a disruption of mTOR-dependent macroautophagy^9^. The contribution of mTORC1-mediated axonal branching to the white matter abnormalities reported in individuals with ASD^17^,^42^,^43^ will be an interesting area of future research.

This study has focused on connectivity between the mPFC and BLA, as this circuit is implicated in ASD pathophysiology and has relevance for the three-chamber social approach deficit observed in germ-line *Pten*^*+/−*^ mice. However, we anticipate that altered connectivity is unlikely to be restricted to this circuit, given that we have observed p-S6 enrichment and neuronal overgrowth outside of the prefrontal cortex (for example, in layer V neurons in the somatosensory cortex). Given the relatively selective behavioural deficits in germ-line *Pten*^*+/−*^ mice^20^, we predict that the behavioural profile of these animals is a product of altered ‘hard-wired' connectivity across multiple circuits, together with the capacity of specific circuits to adapt to these changes during development via synaptic plasticity mechanisms. Indeed, we have found that dopaminergic neurons are enriched for p-S6 during development (Supplementary Fig. 1D). Considering that dopaminergic neurons in the ventral tegmental area encode and modulate features of social interaction^44^, dopamine signalling plays a key neuromodulatory role in the PFC and limbic system^45^,^46^, and conditional deletion of *Pten* in dopaminergic neurons results in three-chamber social approach deficits^20^, we speculate that mesocorticolimbic circuitry may be especially vulnerable to the effects of abnormal structural and functional connectivity resulting from *Pten* haploinsufficiency.

We note that both increased and decreased brain network connectivity has been reported in individuals with ASD^47^. While we have found evidence for hyperconnectivity in our model of macrocephaly/autism syndrome caused by germ-line heterozygous *PTEN* mutations, it is likely that distinct subgroups of individuals with ASD may display different connectivity endophenotypes that correlate with underlying risk factors. It is possible that either increased or decreased connectivity may cause similar behavioural and cognitive deficits, potentially resulting from imbalanced excitation and inhibition^48^ across brain circuits and areas critical for processing of social information.

An important problem for developing therapeutic approaches to psychiatric disorders is defining critical periods of phenotypic reversibility. Previous studies have shown that the mTORC1 inhibitor rapamycin, when given to *Pten* conditional knockout animals at different time points, is able to prevent or arrest, but not reverse, altered neurite projection patterns^10^,^49^. Our results show that suppression of S6K1 by PF-4708671 during early postnatal life, but not in adulthood, results in a lasting correction of mPFC–BLA hyperconnectivity, excessive c-fos reactivity to social cues, and three-chamber social approach deficits in *Pten*^*+/−*^ mice. This indicates that suppression of mTOR-S6K signalling in adulthood is not sufficient to reverse structural connectivity changes (i.e. hyperconnectivity of mPFC–BLA circuitry) in *Pten*^*+/−*^ mice. Our experimental design included a washout period for PF-4708671 because we wanted to test whether treatment with this drug during development or in adulthood results in lasting changes in structural connectivity. We cannot exclude the possibility that overlapping PF-4708671 treatment with behavioural testing may result in modification of behavioural deficits through a mechanism independent of changes in structural connectivity.

While our data indicate that abnormal mPFC–BLA structural connectivity is hard-wired in adulthood, we find that reducing activity in mPFC–BLA projections via DREADD technology is capable of normalizing both c-fos reactivity and the three-chamber social approach deficits. Thus, we propose that there are at least two distinct windows and strategies whereby social behavioural deficits in *Pten*^*+/−*^ mice can be modulated. The first window is in early postnatal life, when suppression of mTORC1-S6K1 can prevent or arrest the development of altered mPFC–BLA structural connectivity. The second window is in adulthood, where modulation of activity in mPFC–BLA circuitry can offset the consequences of pathological structural and functional connectivity. Future work will explore the specific nature of altered structural and functional connectivity between the mPFC and BLA at the level of cell types within the BLA in *Pten*^*+/−*^ mice and whether modulating activity of this circuit is capable of influencing social behaviour in WT animals. It will also be interesting to investigate whether the modulation of amygdala reactivity, habituation and connectivity with the prefrontal cortex that has been associated with genetic variants influencing serotonin signalling^50^,^51^,^52^ might be a substrate for modification of social behavioural endophenotypes in *Pten*^*+/−*^ mice by second-site mutations in serotonin transporter^21^ and pharmacological targeting of serotonin receptor signalling^22^.

An emerging theme in the field of psychiatric disorders is a shift towards stratifying populations based on common underlying risk factors and pathophysiology, and aligning preclinical research to investigate circuit and neurophysiological correlates of these risk factors^53^. We have shown that haploinsufficiency for *Pten* leads to dysregulation of a disease-implicated signalling pathway (the mTOR signalling pathway) that regulates neuronal connectivity, and altered connectivity in an autism-relevant neural circuit (mPFC-amygdala) affects activity in a key brain region (amygdala) in response to social cue, as well as social behaviour. Given that both mPFC–BLA connectivity and mTOR-S6K signalling are highly evolutionarily conserved between rodents and humans^25^,^54^, we anticipate that the mechanism of pathogenesis and corresponding therapeutic strategy presented here may be relevant for individuals with ASD who have been exposed to risk factors associated with dysregulated mTOR signalling.

## Methods

### Mice

*B6.129-Pten*^*tm1Rps*^ (*Pten*^*+/−*^)^55^ were obtained from the repository at the National Cancer Institute at Frederick, where they were already backcrossed onto a congenic C57BL/6J background by the Donating Investigator. The line has been maintained by backcrossing to C57BL/6J mice for more than 10 generations. All other mouse lines were obtained from the Jackson Laboratory: *Etv1*^*tm1.1 (cre/ERT2)Zjh*^(*Etv1-CreERT2*, stock number 013048), *Gt (ROSA)26Sor*^*tm14 (CAG−tdTomato)Hze*^ (*Ai14*, stock number 007914), *Rptor*^*tm1.1Dmsa*^(*Rptor*^loxp/loxp^, stock number 013188), *Emx1*^*tm1 (cre)Krj*^ (*Emx1-Cre*^*+*^, stock number 005628), *Pten*^*tm1Hwu*^(*Pten*^*loxP/loxP*^, stock number 006440), and *Fmr1*^*tm1Cgr*^ (*Fmr1*^*−/y*^, stock number 003025). To obtain *Emx1-Cre*^*+*^; *Rptor*^*loxp/+*^; *Pten*^*+/−*^mice, *Rptor*^*loxp/loxp*^; *Pten*^*+/−*^ male mice were mated with *Emx1-Cre*^*+*^ female mice. *Rptor*^*loxp/+*^ mice were used as controls. All animal experiments were conducted in accordance with NIH and AAALAC guidelines and were approved by The Scripps Research Institute's Institutional Animal Care and Use Committee.

### Immunohistochemistry

Mouse brains were fixed in 4% PFA, incubated in a 20% sucrose/PBS solution at 4 °C for 2 days, and embedded in Tissue-Tek OCT compound (Sakura). Coronal or sagittal sections were collected on Superfrost/Plus slides and immunostained with the following antibodies. Primary antibodies used in this study include phospho-S6 (Ser235/236; 1:2,000, Cell Signaling, #4858), CTIP2 (1:2,000, Abcam, ab18465), SATB2 (1:500, Abcam, ab51502) and c-fos (1:1,000, Millipore, ABE457). Alexa Fluor 488, 594 and 647 conjugated secondary antibodies (Life Technologies) were used in this study. Immunofluorescent brain sections were mounted and counterstained with VectaShield with DAPI (Vector Laboratories, H-1500). For c-fos staining, ABC (avidin-biotin complex) kit (Vector Laboratories, PK-6101) and DAB (3,3′ Diaminobenzidine; Life Technologies, #34001) were used. Images were obtained with a Olympus VS120 microscope and processed using the VS-DESKTOP software (Olympus).

c-fos immunoreactive neurons were quantified manually using the VS-DESKTOP software (Olympus) in the mPFC, including the prelimbic and infralimbic cortices (PL and IL; bregma 1.98, 1.94 and 1.78 mm), and in the BLA (bregma −1.46, −1.58 and −1.70 mm) of coronal sections. For each region, the number of c-fos^+^ neurons was quantified in three coronal brain sections from both left and right hemispheres, and averaged within each animal. The c-fos activation index was calculated as the average number of c-fos^+^ neurons in each region divided by the time that the mouse spent sniffing the novel mouse during the social exposure period.

### Cell size analysis

Brain sections were stained with NeuroTrace green fluorescent Nissl stain (1:100 dilution). Regions of interest (ROIs) were cropped from primary somatosensory cortex barrel field (coronal sections) using Photoshop (Adobe). Each layer (layers II–VI) was further cropped according the anatomical boundaries as judged by fluorescent Nissl stain. Cell soma size of all neurons in each layer was measured^56^ using ImageJ (NIH). For BLA-projecting mPFC neurons, mCherry^+^ cells were measured. The freehand tool in ImageJ was used to contour the boundary of neuronal soma. Three ROIs were measured in each animal.

### Western blot analysis

Whole cerebral corticies or prefrontal cortices were extracted from mice, snap frozen in liquid nitrogen and stored at −80 °C until used. Tissue was homogenized in radioimmunoprecipitation buffer containing the Protease Inhibitor Cocktail (Roche) and phosphatase inhibitors (Sigma-Aldrich). Total protein concentrations were measured using the Pierce BCA Protein Assay Kit (ThermoScientific), and samples were diluted for equal amounts of protein in each lane. Proteins were electrophoresed onto NuPAGE 4–12% Bis-Tris Gel (Novex, Life Technologies) and were subsequently transferred to polyvinylidene difluoride membranes (Immobilon, Millipore). Primary antibodies used in this study were phospho-S6 (S235/236; 1:2,000, Cell Signaling, #4858), S6 (1:2,000, cell signaling, #2217), CRMP2 (1:1,000, Cell Signaling, #9393), Tau (1:1,000, Cell Signaling, #4019) and β-actin (1:2,000, Cell Signaling, #4970). Peroxydase-conjugated anti-rabbit IgG secondary antibody (1:5,000, Jackson ImmunoResearch Laboratories, Inc.) was used. Proteins were visualized with enhanced chemiluminescence using the WesternBright Quantum kit (Advansta) and quantified using ImageJ software. Phospho-S6 level was normalized to total S6 level. Graphs represent an average of at least 5 animals. Images have been cropped for presentation. Full size images are presented in Supplementary Figs 8 and 9.

### Pharmacological S6K1 inhibitor treatment

The S6K1 inhibitor PF-4708671 was dissolved in DMSO. Both vehicle and PF-4708671 were formulated in 10% DMSO, 10% tween 80 and 80% water. PF-4708671 (75 mg kg^−1^) or vehicle were injected subcutaneously into pups from P4 to P14. For adult female mice (2–4 month of age), intraperitoneal injection of PF-4708671 (75 mg kg^−1^) was performed for 11 consecutive days. 75 mg kg^−1^ was selected based on prior studies reporting that doses of PF-4708671 in this range can suppress p-S6 levels in the adult mouse brain^57^.

### 3D neuronal reconstruction

*Etv1-CreERT2*^*+*^*; Ai14*^*+/−*^*; Pten*^*+/−*^ and *Etv1-CreERT2*^*+*^*; Ai14*^*+/−*^*; Fmr1*^*−/y*^ mice, as well as controls, received subcutaneous tamoxifen injections (3 mg kg^−1^) at P7, and were perfused at P14. Brains were fixed in 4% PFA, incubated in a 20% sucrose/PBS solution at 4 °C for 3 days, and embedded in Tissue-Tek OCT compound. Coronal sections (500 μm thickness) in the primary somatosensory cortex were collected and incubated in Scale A2 solution^58^ for at least 14 days, making the tissue transparent. Layer V neurons were imaged using a Confocal Laser Scanning Microscope Leica TCS SP8, and reconstructed and analysed using Neuromantic reconstruction software^59^, with Sholl analysis performed using Neurolucida.

### Axon tracing of mPFC to BLA projections

Axon tracing in this study followed a previously published method^60^. AAV-tdTomato was obtained from Penn Vector Core. For injections, mice were anaesthetized under isoflurane and placed onto a stereotaxic frame. Bregma and lambda were used to find the coordinates of the mPFC (1.9 mm anterior to bregma, 0.4 mm lateral to midline and 2.3 mm ventral to bregma). The virus, diluted to 10% with water, was injected by a syringe pump at a rate of 100 nl min^−1^, with 200 nl volume injected per site in each mouse. Mice were perfused 2 weeks later, allowing for the expression of tdTomato. 100 μm-thick sagittal brain sections were collected to image mPFC axon terminals in the BLA (based on mouse brain atlas sagittal sections from the Allen Brain Atlas), with setting as z-series (stacks at 1 μm depth interval) using a × 25 Leica objective with constant parameter for laser power and gain. tdTomato expressing axons (300–350 μm length including the axon terminal) in the BLA were traced and analysed using Neuromantic reconstruction software^59^. Protrusions more than 6 μm long were characterized as axon branches. Boutons were defined as at least two times wider than the width of axons, with fluorescence intensity at least two times that in axon backbones.

### Mouse behaviour tests

For three-chamber social approach test, mice were tested as previously described^20^,^21^. Briefly, mice were housed under a reversed 12:12 h light cycle for at least 2 weeks before testing. Mice were put into the apparatus for 5 min to habituate on each of the 2 days preceding the test, and then returned to the housing room. During the test, each mouse was put into the three-chamber apparatus for 5 min for habituation, followed by 10 min of testing with a novel stimulus mouse inside a tube of one chamber and an empty tube in the chamber of the other side (the centre chamber was left empty). The per cent time spent in each of the three chambers, containing either a stimulus mouse in a tube, an empty tube or nothing (in the centre) was recorded during the 10-min social approach trial.

For non-matching to sample T-maze task, mice were food restricted to 85–90% of *ad libitum* body weight, to increase motivation for this test. The behaviour protocol consisted of 3 days of habituation, followed by alternation training and then delay testing. During habituation, mice were allowed to explore the maze for 4 min per trial, with a total of 4 trials per day. All mice learned to obtain food reward from the trough after 3 days of habituation. During the alternation training, each trial consisted of one forced and one free-choice run. In the forced run, the mouse ran down the centre arm of the maze and was directed into one of the arms. The mouse was then returned to the start box. In the free-choice run, the mouse was required to select the arm opposite to that visited in the forced run to receive a food reward. Mice were given daily training of four trials until they reached criterion, defined as performance of at least 75% correct per day for 2 consecutive days. Both WT and *Pten*^*+/−*^ mice reached criterion after 7 days of training. During delay testing, each day consisted of two non-delay trials and two delay trials. During delay trials, the mouse was kept in the start box for 60 s between the forced and free-choice runs. Mice received 5 days of delay testing, for a total of 10 trials each of delay and non-delay testing. The non-delay tests were used to determine if the mice maintained criterion performance.

For social exposure without fixed interaction time, female mice were housed individually in a home cage containing a perforated acrylic tube for 7 days before the test. On the test day, a novel conspecific female stimulus mouse was placed in the tube within the home cage of subject female mice. The subject mouse was allowed to explore the stimulus mouse for 20 min (social exposed group) and then the stimulus mouse was removed. The behaviour was video-recorded and manually scored to determine the amount of time that the subject mouse spent sniffing the stimulus mouse. After the stimulus mouse was removed, the subject mouse remained in the home cage for an additional 2 h before being perfused. For the control group, mice were individually housed in a home cage containing a perforated acrylic tube for 7 days and then perfused.

For social exposure with fixed interaction time, female mice were housed individually in a home cage containing a perforated acrylic tube for 7 days before the test. On the test day, a novel conspecific female stimulus mouse was placed in the tube within the home cage of subject female mice. The subject mouse was allowed to explore the stimulus mouse until it reached a fixed criterion of 30 s of sniffing time (manually scored during the social exposure period). In this way, each subject mouse received roughly equivalent social cue exposure.

### DREADD experiments

Mice were anaesthetized with isoflurane in the stereotaxic frame for the entire surgery and their body temperature was maintained with a heating pad. To reduce activity in mPFC neurons projecting to the BLA, 350 nl of canine adenovirus carrying Cre recombinase (CAV2-Cre)^61^,^62^ was bilaterally injected into the BLA (1.45 mm posterior to bregma, 3.3 mm lateral to midline and 5.2 mm ventral to bregma), and 400 nl of AAV carrying Cre-dependent hM4Di and mCherry (AAV-DIO-hM4Di-mCherry) or mCherry alone (AAV-DIO-mCherry) were bilaterally injected into mPFC (1.9 mm anterior to bregma, 0.4 mm lateral to midline, and 2.3 mm ventral to bregma). Injections were performed at a rate of 50 nl per minute. The needle was allowed to sit in the target location for 3 min before the start of viral injection and for 7 min after injection was complete. Social behaviour was tested 4 and 6 weeks after the surgery, with each mouse being tested following vehicle and CNO injections in a counterbalanced design. Mice were given i.p. injections of either vehicle or CNO (3 mg kg^−1^ body weight) 30 min before social approach test. CNO was dissolved in saline to a working solution of 0.5 mg ml^−1^.

### Laminar plot analysis

After image acquisition, ROIs were cropped from the primary somatosensory cortex across the radial thickness using Photoshop. Cerebral cortex thickness was normalized, and images were then auto-adjusted for each channel and saved. Plot profiles for each channel of each image were obtained using ImageJ. Three brains of WT mice were analysed, and 3 ROIs per brain were used for the laminar plot analysis.

### SUnSET method for measurement of protein synthesis

We obtained 400 μm coronal prefrontal cortical slices from postnatal day 8 (P8) WT and *Pten*^*+/−*^ mice. Slices were allowed to recover in artificial cerebrospinal fluid (in mM: 124 NaCl, 2.8 KCl, 2 CaCl_2_, 1.25 NaH_2_PO_4_, 1.25 MgSO_4_, 26 NaHCO_3_, 10 Dextrose) for 2 h at 32 °C. Puromycin (5 μg ml^−1^) was applied to slices for 40 min at 32 °C to label newly synthesized proteins. Slices were then snap frozen on dry ice and stored at −80 °C until used. Protein (30 μg) was loaded on 4–12% gradient gels, transferred onto polyvinylidene difluoride membranes, and probed with anti-puromycin antibody (1:5,000, Millipore, MABE343). Anti-mouse HRP-tagged secondary antibody was used and chemiluminiscence was developed using standard ECL reagents. Slices that were not exposed to puromycin were used to control for non-specific labelling. Quantification was done by measuring intensity of each lane from 14 to 191 kDa, subtracting it from the intensity of the unlabelled lane, normalized to loading control and expressed relative to WT.

### Statistical analysis

Prior studies using similar experiments or power analysis were used to determine sample size. Planned comparisons between WT and mutant mice were performed for all assays using independent-sample *t* tests. One-way analyses of variance (ANOVAs) were used to assess genotype effect in experiments containing control*, Pten*^*+/−*^, *Emx1-Cre*^*+*^; *Rptor*^*loxp/+*^, and *Emx1-Cre*^*+*^; *Rptor*^*loxp/+*^; *Pten*^*+/−*^ mice. Tukey's *post hoc* tests were used where appropriate. Two-way ANOVAs were used to assess drug and genotype effects and their interactions in experiments containing WT and *Pten*^*+/−*^ mice with vehicle or PF-4708671 treatment; Tukey's *post hoc* tests were used whereappropriate. Planned comparison independent-sample *t* tests compared WT and *Pten*^*+/−*^ with vehicle treatment, and compared vehicle and PF-4708671-treated *Pten*^*+/−*^ mice. For the three-chamber social approach test, two-way ANOVAs were used to assess the interaction between chamber time and genotype; Sidak's *post hoc* tests were used where appropriate. Paired *t* tests were also used within genotype to determine if mice showed a preference for the social chamber. All statistics were performed using Graphpad, with significance set at *P*<0.05. Throughout the manuscript, values represent means; error bars indicate s.e.m.'s; *N* values refer to biological replicates. All measurements and testing were performed blind to the genotype or experimental manipulations.

### Data availability

All relevant data are available upon request.

## Additional information

**How to cite this article:** Huang, W.-C.H. *et al.* Hyperconnectivity of prefrontal cortex to amygdala projections in a mouse model of macrocephaly/autism syndrome. *Nat. Commun.* **7,** 13421 doi: 10.1038/ncomms13421 (2016).

## Supplementary Material

Supplementary Information Supplementary Figures 1-9.
