# Supplementary material for: Hyperconnectivity of prefrontal cortex to amygdala projections in a mouse model of macrocephaly/autism syndrome
Source: Nat Commun. 2016 Nov 15;7:13421. doi: 10.1038/ncomms13421 (PMC5116076; doi:10.1038/ncomms13421)
Supplement: Supplementary Information — Supplementary Figures 1-9. [file ncomms13421-s1.pdf]

# Supplemental Figure 1

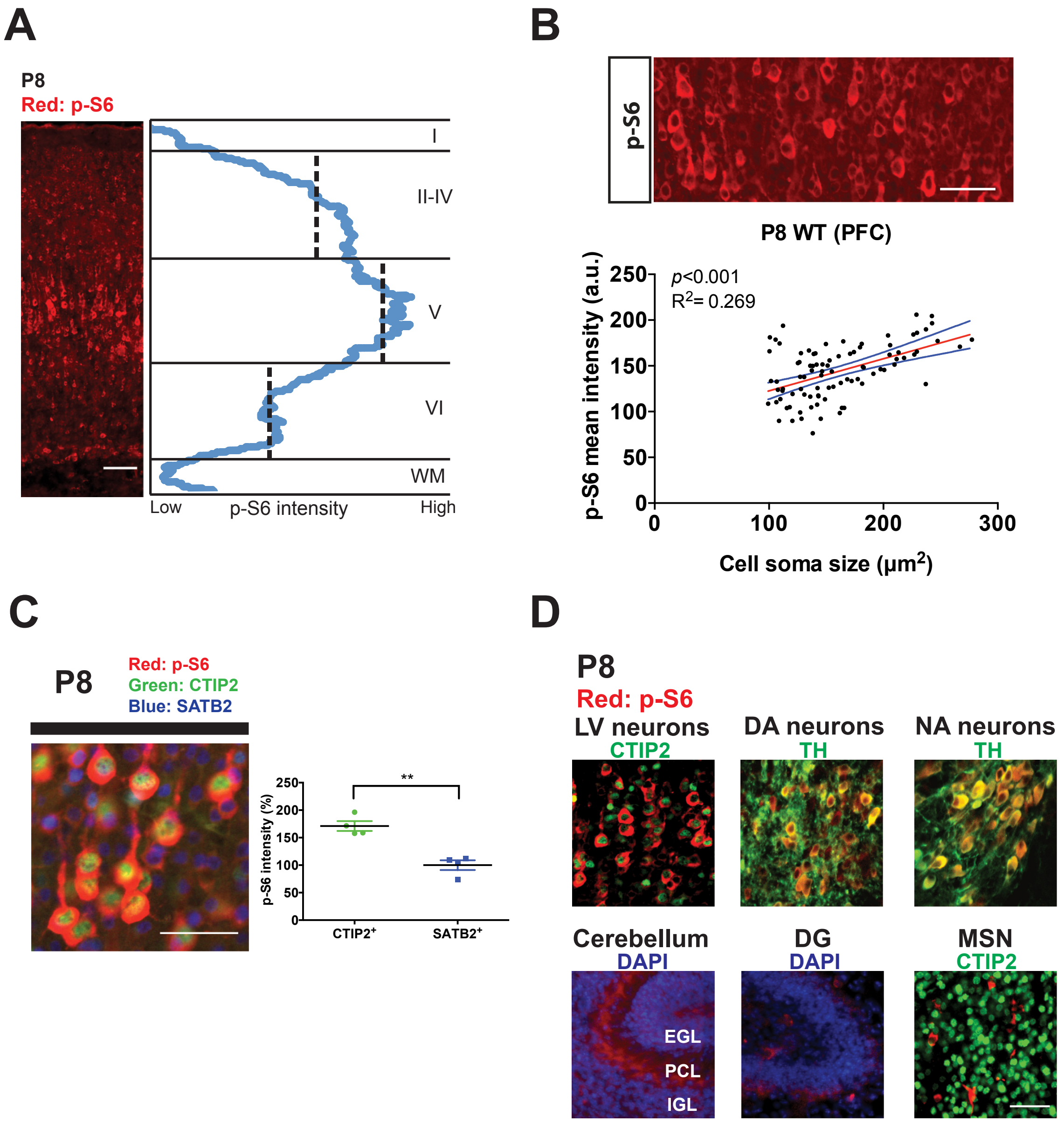

**Supplementary Figure 1 Developmental mTORC1 activity and cell size in the developing mouse brain.**

(A) IHC for phospho-S6, a readout for mTORC1 activity, (p-S6, red) in P8 wild type mouse cerebral cortex, and laminar plot for p-S6 immunoreactivity. Scale bar: 100 μm. (B) Representative images of p-S6 staining in the prefrontal cortex (PFC) of P8 wild type mice. Phospho-S6 immunoreactivity is correlated with cell soma size at P8. N=90 neurons/5 animals. Scale bar: 50 μm. (C) IHC for p-S6 (red), CTIP2 (green), and SATB2 (blue) in the layer V cortex of P8 wild type mice. Scale bar: 50 μm. Independent-sample t-tests were used. ** $p<0.01$. N=4 animals. (D) IHC for p-S6 (red) with CTIP2 (green), markers for layer V (LV) neurons in the cerebral cortex or medium spiny neurons (MSN) in the striatum, tyrosine hydroxylase (TH) (green), markers for dopaminergic (DA) neurons in ventral tegmental area or noradrenergic (NA) neurons in locus coeruleus, DAPI (blue) in cerebellum and hippocampus dentate gyrus (DG) in postnatal day 8 (P8) mouse brain. EGL: external granule layer, PCL: Purkinje cell layer, IGL: internal granule layer. Scale bar: 50 μm. All mice used in this figure were male.

# Supplemental Figure 2

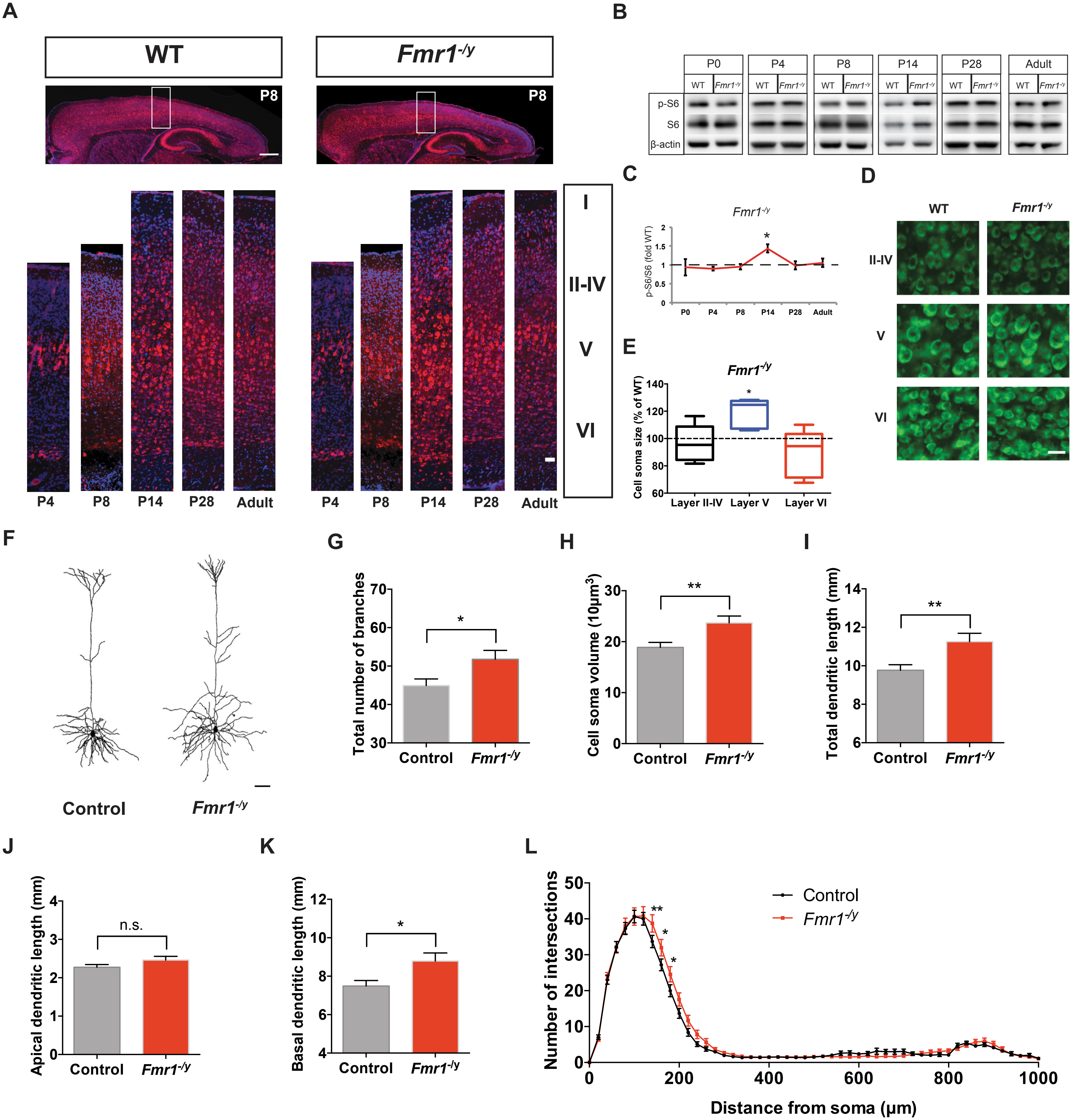

**Supplementary Figure 2 Characterization of mTORC1 activity and neuronal growth in *Fmr1* KO mice.** (A) Images showing phospho-S6 (p-S6), a readout for mTORC1 activity, staining in the cerebral cortex of postnatal day 8 (P8) wild-type (WT) and *Fmr1*$^{-/y}$ mice. Phospho-S6 (red), DAPI (blue), and scale bar: 500 µm. Representative images of somatosensory cortex with p-S6 staining in P4, P8, P14, P28, and adult WT and *Fmr1*$^{-/y}$ mice. Phospho-S6 (red), DAPI (blue), and scale bar: 50 µm. (B) Representative images of Western blot for p-S6, total S6, and β-actin of WT and *Fmr1*$^{-/y}$ cerebral cortex at P0, P4, P8, P14, P28, and adulthood. (C) Quantification of p-S6 levels relative to total S6, expressed as fold WT, in the cerebral cortex of WT and *Fmr1*$^{-/y}$ mice at P0, P4, P8, P14, P28, and adult. Independent-sample t-tests were used. $*p<0.05$. N=5 animals per genotype in each time points. (D) Representative images of fluorescent Nissl stain in layers II-IV, V, and VI somatosensory cortex of WT and *Fmr1*$^{-/y}$ mice at P14. Scale bar: 25 µm. (E) Quantification of cell soma size in layers II-IV, V, and VI somatosensory cortex of WT and *Fmr1*$^{-/y}$ mice at P14, expressed as percent of WT. Independent-sample t-tests were used. $*p<0.05$. N=6 animals in each genotype. (F) Representative images of reconstructed layer V neurons in the somatosensory cortex of P14 *Etv1-CreERT2; Ai14* (Control) and *Etv1-CreERT2; Ai14; Fmr1*$^{-/y}$ (*Fmr1*$^{-/y}$) mice. Scale bar: 50 µm. (G-K) Quantification of total number of branches (G), cell soma volume (H), and total (I), apical (J), and basal (K) dendrite length of layer V neurons in the somatosensory cortex of P14 Control and *Fmr1*$^{-/y}$ mice. Independent-sample t-tests were used. $*p<0.05$, $**p<0.01$, and n.s. indicates no significant difference. N=25 neurons/6 animals each genotype. (L) Sholl analysis of somatosensory cortex layer V neurons in P14 control and *Fmr1*$^{-/y}$ mice. Two-way ANOVA with Bonferroni correction, $F(50, 2448)=1.377$, $p<0.05$ (interaction); $*p<0.05$, and $**p<0.01$. N=25 neurons/6 animals each genotype. All mice used in this figure were male.

# Supplemental Figure 3

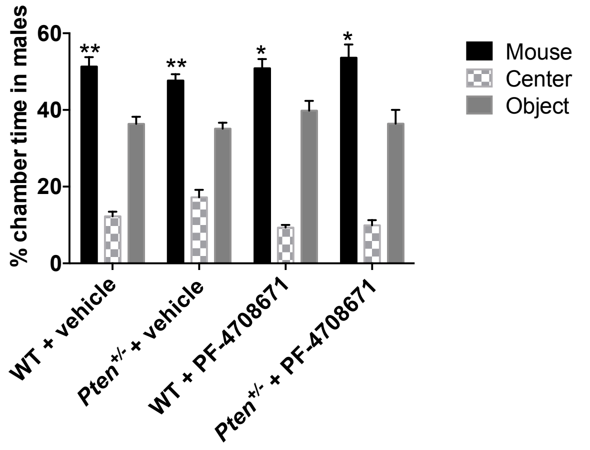

**Supplementary Figure 3 Male *Pten* mutant mice show normal performance in the three chamber social approach task.** Percent time male WT and *Pten*$^{+/-}$ mice receiving either vehicle or PF-4708671 from P4 to P14 spent in each chamber during the 3-chamber social approach test. Two-way ANOVA was used. $F(3,68)=0.5724$, $p=0.6351$ (interaction), $F(3,68)=1.056$, $p=0.3735$ (genotype), and $F(1,68)=62.27$, $p<0.001$ (chamber time). Paired t-test showed that all four groups exhibited a preference for the social chamber. *$p<0.05$ and **$p<0.01$ N= 10 WT receiving vehicle, 9 *Pten*$^{+/-}$ receiving vehicle, 12 WT receiving PF-4708671, and 7 *Pten*$^{+/-}$ receiving PF-4708671.

# Supplemental Figure 4

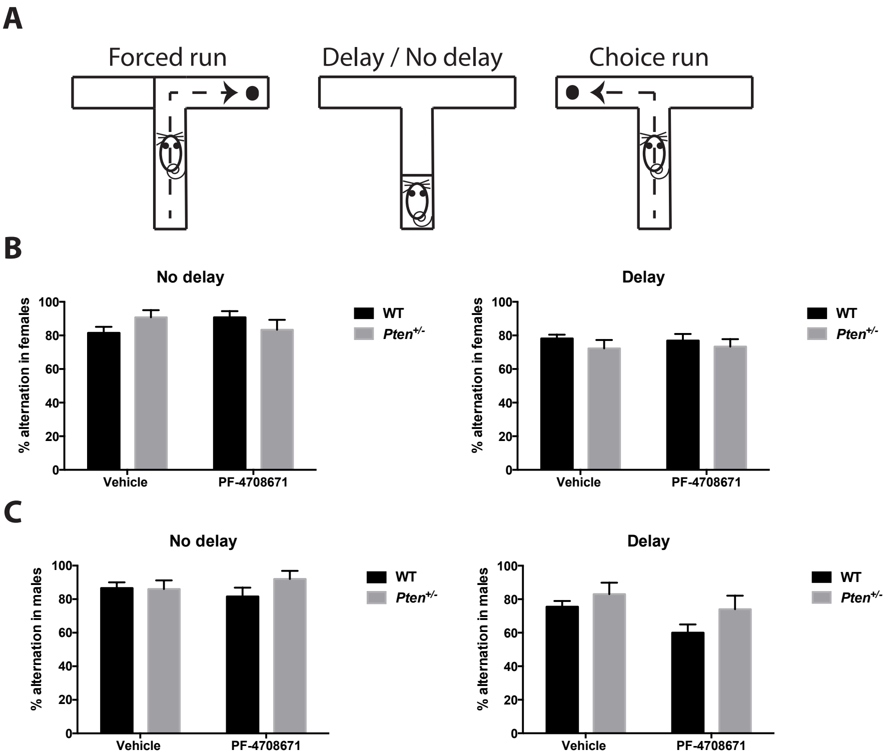

**Supplementary Figure 4 *Pten* mutant mice show normal performance in a spatial working memory task.**

(A) Cartoons illustrating spatial working memory task (see method for detailed description). (B) Percent alternation in female mice with either vehicle or PF-4708671 treatment from P4-P14. N= 13 WT with vehicle treatment, 13 *Pten*$^{+/-}$ with vehicle treatment, 13 WT with PF-4708671 treatment, and 12 *Pten*$^{+/-}$ with PF-4708671 treatment. (C) Percent alternation in male mice with either vehicle or PF-4708671 treatment from P4-P14. N= 9 WT with vehicle treatment, 10 *Pten*$^{+/-}$ with vehicle treatment, 12 WT with PF-4708671 treatment, and 5 *Pten*$^{+/-}$ with PF-4708671 treatment. All mice showed normal performance in both the delay and the no delay tests.

# Supplemental Figure 5

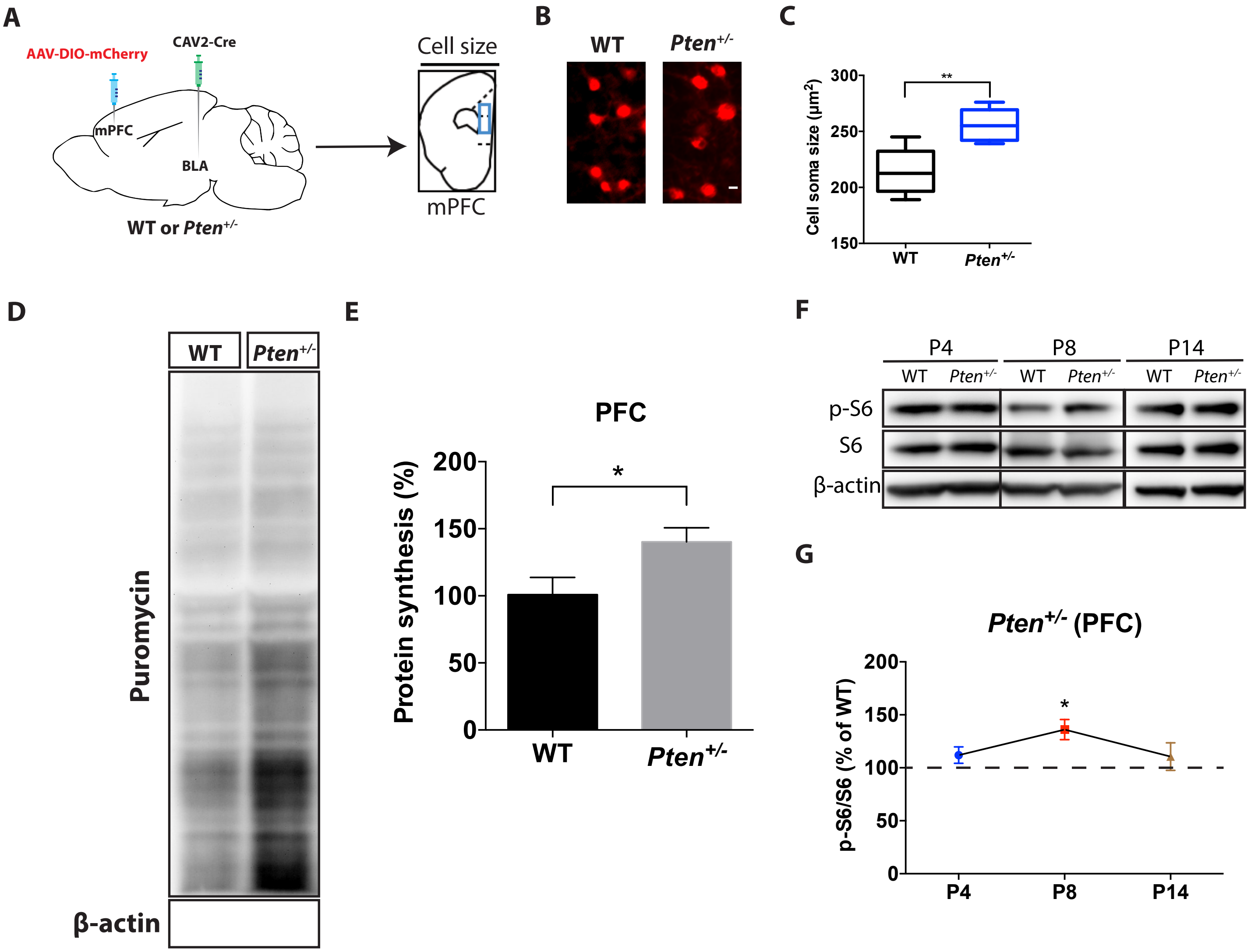

**Supplementary Figure 5 Characterization of mTORC1 activity and neuronal growth in the prefrontal cortex of *Pten* mutant mice.**

(A) Schematic diagram showing experimental workflow. Basolateral amygdala (BLA) projecting medial prefrontal cortex (mPFC) neurons were labeled by injection of CAV2-Cre in the BLA and AAV-DIO-mCherry in the mPFC. (B) Representative images of BLA-projecting mPFC neurons in adult wild type (WT) and $Pten^{+/-}$ mice. Scale bar: 10 μm. (C) Quantification of cell soma size of BLA-projecting mPFC neurons in adult WT and $Pten^{+/-}$ mice. Independent-sample t-tests were used. $^{**}p<0.01$. N=6 animals per genotype. (D) Western blot showing puromycin incorporation into PFC slices from postnatal day 8 (P8) WT and $Pten^{+/-}$ mice. (E) Quantification of puromycin incorporation in PFC slices of P8 WT and $Pten^{+/-}$ mice. Independent-sample t-tests were used. $^{*}p<0.05$. N=6 animals per genotype. (F) Western blot for p-S6, total S6, and β-actin in the PFC of P4, P8, and P14 WT and $Pten^{+/-}$ mice. (G) Quantification of p-S6/S6 in the PFC of P4, P8, and P14 WT and $Pten^{+/-}$ mice. Graph presented as percent of WT. Independent-sample t-tests were used. $^{*}p<0.05$. N=6 animals per genotype each time point. All mice used in this figure were female.

# Supplemental Figure 6

**A.**

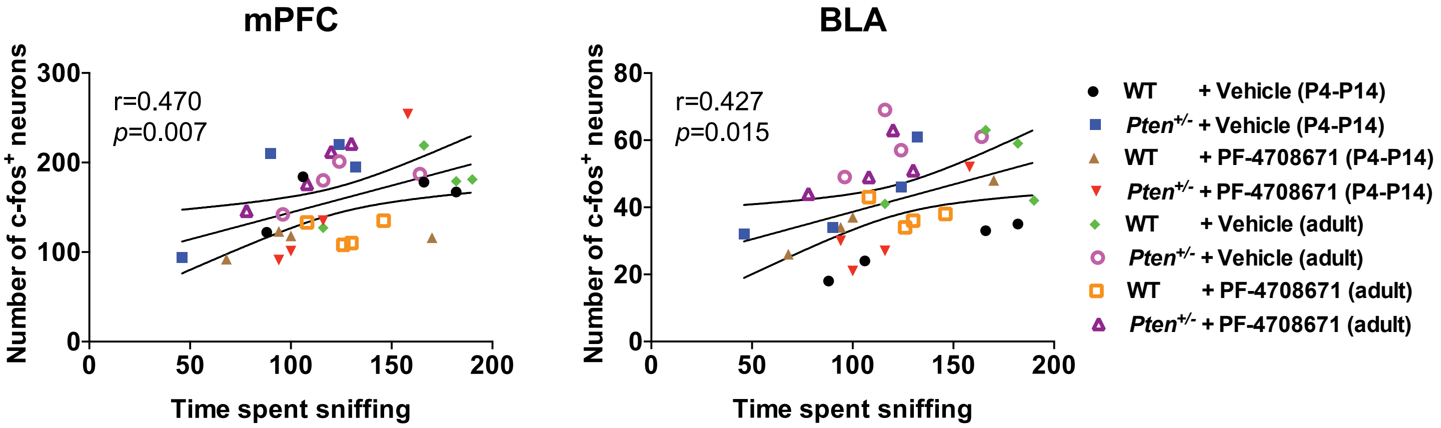


**B**

$$\text{c-fos activation index} = \frac{\text{c-fos}^{+}\text{ neuron counts}}{\text{Time spent sniffing}}$$

**Supplementary Figure 6 c-fos cell number in the mPFC and BLA is correlated with social interaction time.**

(A) Graph showing a positive correlation of c-fos$^+$ neurons in mPFC or BLA with time spent sniffing. N=32 animals, a combination of 4 animals in each group (WT and *Pten*$^{+/-}$ mice receiving either vehicle or PF-4708671 from P4 to P14 or in adulthood). (B) Formula showing the calculation of c-fos activation index. All mice used in this figure were female.

# Supplemental figure 7

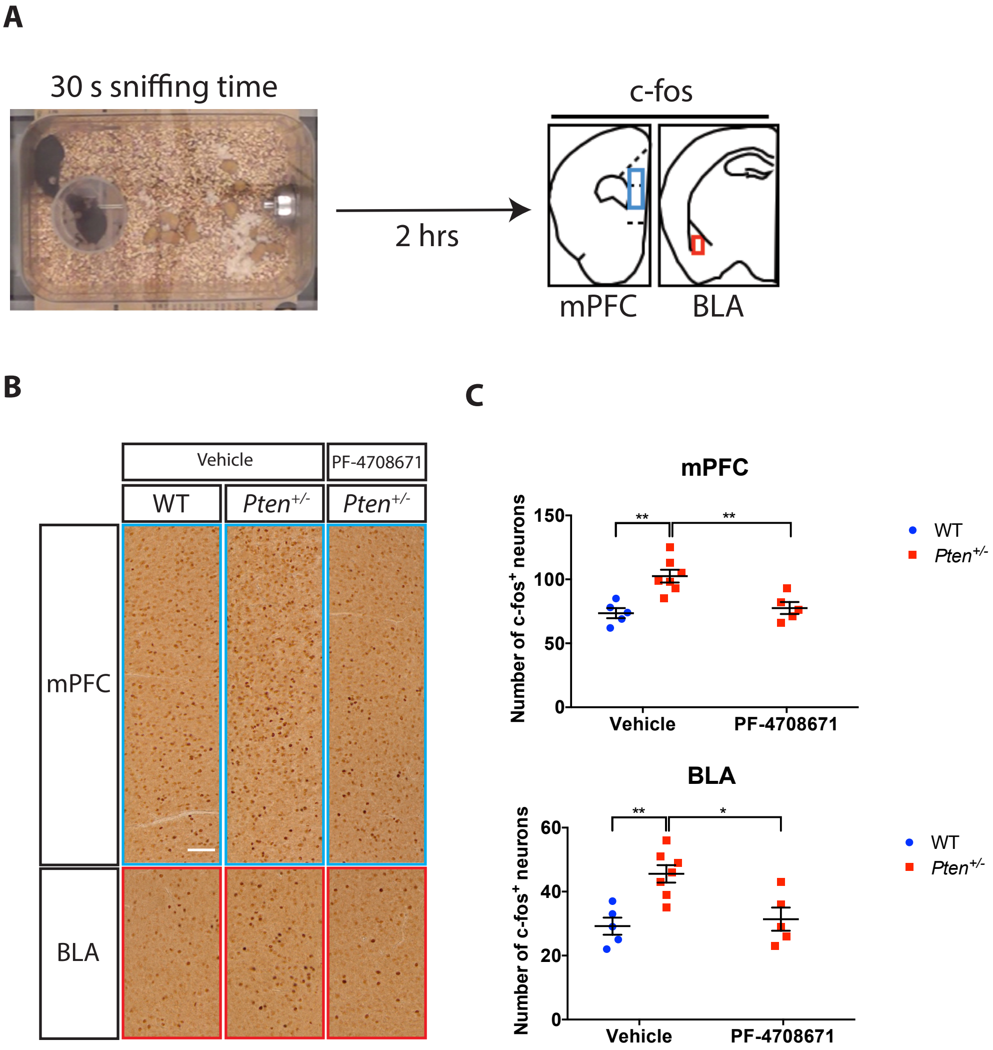

**Supplementary Figure 7 Increased c-fos cell number in the mPFC and BLA of *Pten* mutant mice.**

(A) Diagram showing the flow of the experiment. Mice were exposed to a novel stimulus mouse until they reached 30 seconds of sniffing time. Mice were perfused and brains were extracted for c-fos staining 2 hours after the social exposure. (B) IHC for c-fos in the mPFC and BLA of WT and $Pten^{+/-}$ mice with either vehicle or PF-4708671 injection from P4 to P14. Scale bar: 100 μm. (C) Quantification of c-$fos^{+}$ cells in the mPFC and BLA of WT and $Pten^{+/-}$ mice with either vehicle or PF-4708671 injection from P4 to P14. Planned comparisons revealed a significant difference between WT and $Pten^{+/-}$ mice receiving vehicle, and a significant difference between vehicle and PF-4708671 injected $Pten^{+/-}$ mice. *$p$<0.05, and **$p$<0.01. N=5 animals in vehicle injected WT mice, 7 animals in vehicle injected $Pten^{+/-}$ mice, and 5 animals in PF-4708671 injected $Pten^{+/-}$ mice. All mice used in this figure were female.

# Supplementary Figure 8

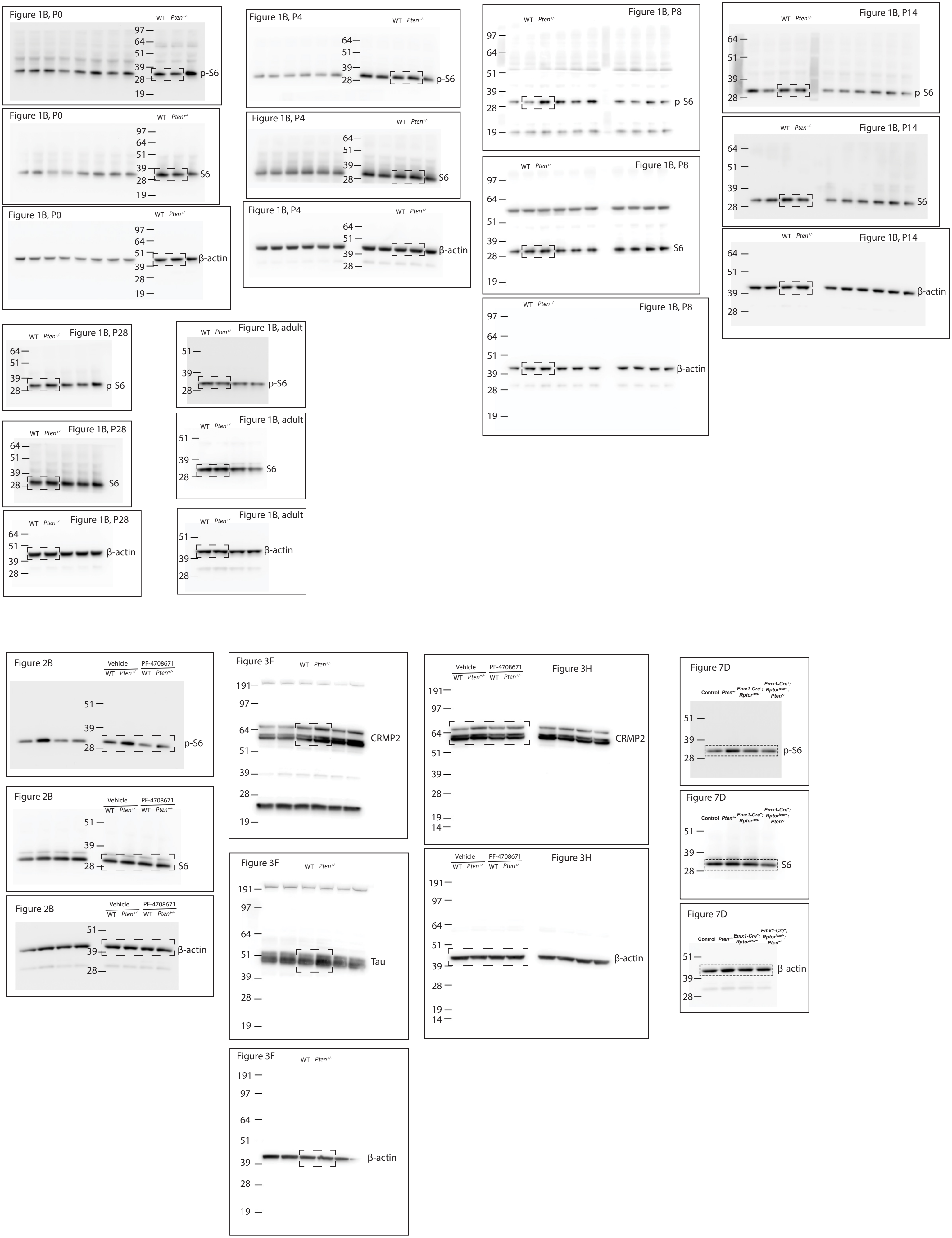

**Supplementary Figure 8 Full size Western blot images.** Full size Western blot images for figure 1B, 2B, 3F and H, and 7D.

## Supplementary Figure 9

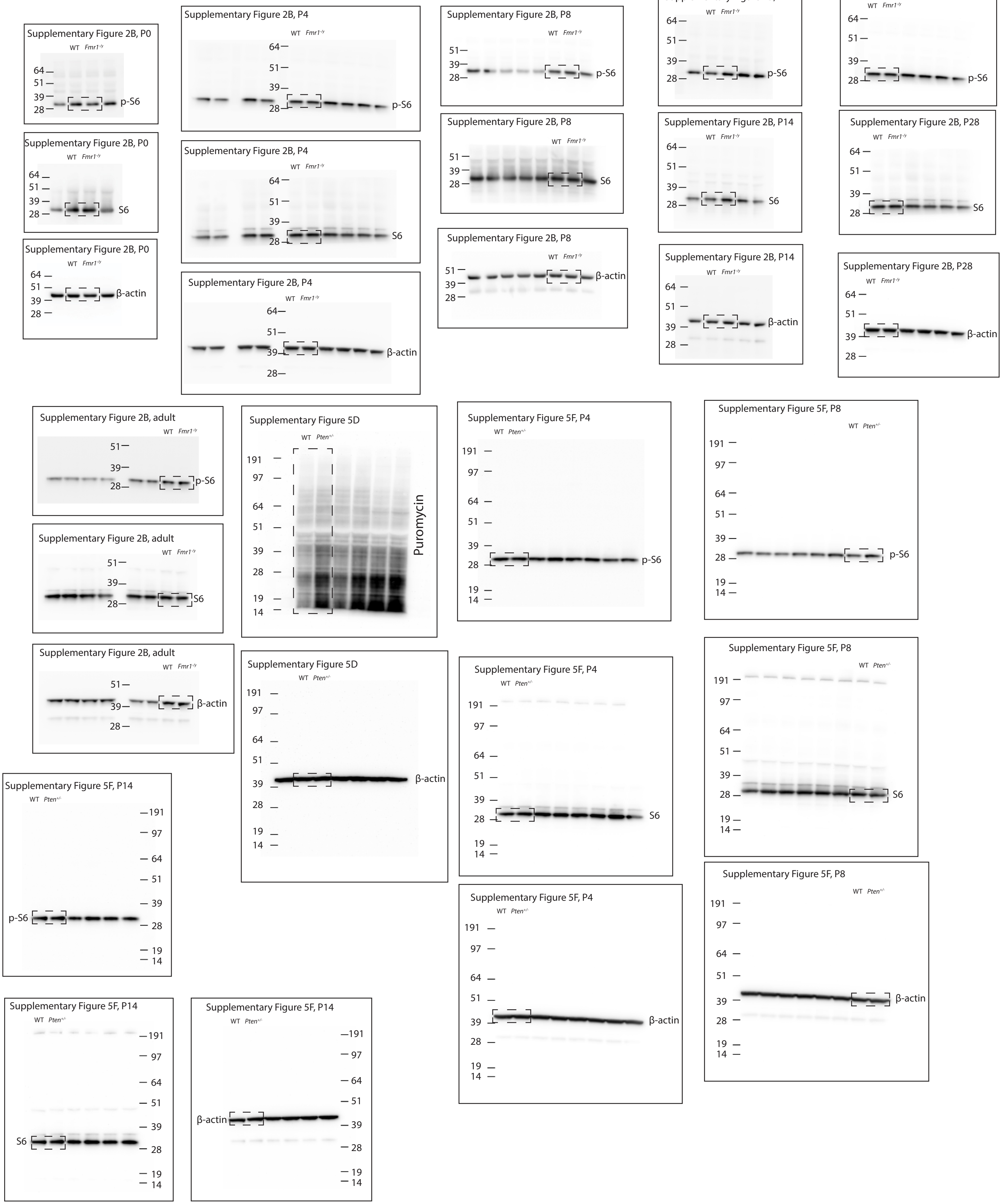

**Supplementary Figure 9 Full size Western blot images.** Full size Western blot images for supplementary figure 2B, 5D and F.
